# Mapping the dynamics and nanoscale organization of synaptic adhesion proteins using monomeric streptavidin

**DOI:** 10.1038/ncomms10773

**Published:** 2016-03-16

**Authors:** Ingrid Chamma, Mathieu Letellier, Corey Butler, Béatrice Tessier, Kok-Hong Lim, Isabel Gauthereau, Daniel Choquet, Jean-Baptiste Sibarita, Sheldon Park, Matthieu Sainlos, Olivier Thoumine

**Affiliations:** 1 Interdisciplinary Institute for Neuroscience, UMR 5297, Centre National de la Recherche Scientifique, 33077 Bordeaux, France; 2 Interdisciplinary Institute for Neuroscience, University of Bordeaux, 33077 Bordeaux, France; 3 Department of Chemical and Biological Engineering, University at Buffalo, Buffalo, New York 14260, USA; 4 Bordeaux Imaging Center, UMS 3420 Centre National de la Recherche Scientifique, University of Bordeaux, US 4 INSERM, 33077 Bordeaux, France; 5 Imagine Optic, 18 rue Charles de Gaulle, 91400 Orsay, France

## Abstract

The advent of super-resolution imaging (SRI) has created a need for optimized labelling strategies. We present a new method relying on fluorophore-conjugated monomeric streptavidin (mSA) to label membrane proteins carrying a short, enzymatically biotinylated tag, compatible with SRI techniques including uPAINT, STED and dSTORM. We demonstrate efficient and specific labelling of target proteins in confined intercellular and organotypic tissues, with reduced steric hindrance and no crosslinking compared with multivalent probes. We use mSA to decipher the dynamics and nanoscale organization of the synaptic adhesion molecules neurexin-1β, neuroligin-1 (Nlg1) and leucine-rich-repeat transmembrane protein 2 (LRRTM2) in a dual-colour configuration with GFP nanobody, and show that these proteins are diffusionally trapped at synapses where they form apposed *trans*-synaptic adhesive structures. Furthermore, Nlg1 is dynamic, disperse and sensitive to synaptic stimulation, whereas LRRTM2 is organized in compact and stable nanodomains. Thus, mSA is a versatile tool to image membrane proteins at high resolution in complex live environments, providing novel information about the nano-organization of biological structures.

## Abstract (web summary)

The advent of fluorescence-based super-resolution microscopy has created a need for labeling strategies relying on small probes that minimally perturb protein function. Here the authors describe a labeling method that reduces protein tag and label sizes, allowing for accurate protein targeting and measurements of protein dynamics in tight cellular spaces.

## Graphical abstract

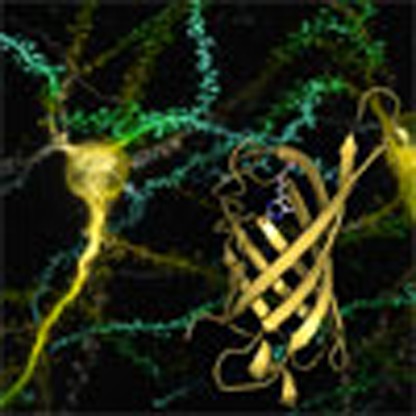

Critical cellular functions including adhesion and signalling are performed by dynamic macromolecular platforms at the cell membrane. Synaptic neuronal contacts are examples of such complex structures, where protein concentration is extremely high and lies in a very confined and compartmentalized space^1^. Recently developed fluorescence-based super-resolution imaging (SRI) techniques, including STimulated Emission Depletion (STED)^2^, PhotoActivation Localization Microscopy (PALM)^3^,^4^, direct STochastic Optical Reconstruction Microscopy (dSTORM)^5^,^6^ and universal Point Accumulation for Imaging in Nanoscale Topography (uPAINT)^7^,^8^, provide 20–50 nm resolution maps of single-molecule localization in biological samples, allowing a better understanding of the organization and turnover of these submicron multisubunit assemblies^9^. To accompany such progress in imaging power, there is a pressing need for efficient labelling strategies relying on small and penetrating probes that provide high signal-to-noise ratio and minimal linkage error with respect to target proteins^10^,^11^.

Protein labelling has relied for a long time on the use of antibodies, which have the strong advantage of targeting endogenous proteins. Yet, full-length antibodies are relatively large (10–15 nm) compared with nanoscale biological objects, thereby causing potential steric hindrance and localization bias^11^,^12^. Moreover, because of their divalence, antibodies may induce protein crosslinking at the cell surface. The recent expansion of antibody fragments such as Fab, scFv or VhH is promising improvements^13^,^14^; however, their development including antigen preparation is time-consuming^15^. Alternative labelling approaches consist of the use of recombinant proteins fused to fluorescent proteins (FPs, photoactivatable or photoswitchable proteins)^3^,^16^, reactive moieties such as modified enzymes to bind small dye-labelled synthetic molecules^17^,^18^ or small peptide tags for subsequent conjugation with exogenous fluorescent ligands^19^,^20^. However, FPs usually have weaker fluorescence compared to organic dyes, and the insertion of a FP or enzyme moiety (25–35 kDa) may affect proper protein folding and/or function^21^,^22^.

In this context, we searched for an efficient and orthogonal labelling approach combining minimal tag size on the protein of interest, probe penetrability in confined regions, absence of artefact due to multivalence and compatibility with multicolour imaging. We propose a new method based on streptavidin monomers (mSA) to deliver bright organic fluorophores to proteins that are enzymatically biotinylated on a 15-amino-acid acceptor peptide (AP) tag^23^. This targeted biotinylation strategy was previously developed in combination with monovalent streptavidin to track membrane molecule dynamics using quantum dots^24^. However, monovalent streptavidin remains a relatively large heterotetramer (molecular weight of 60 kDa and ∼6 nm across) and is difficult to produce because of the precise 3-to-1 stoichiometry that is required among the subunits^24^. mSA is a structural monomer evolved from wild-type streptavidin tetramer with the aim of maximizing aqueous stability (denaturation temperature of 60 °C) while maintaining sufficient biotin affinity (*K*_d_=2.8 nM)^25^,^26^. The resulting mSA is a stable 3-nm labelling probe with a molecular weight of 12.5 kDa, which is simple to produce and to couple to various fluorophores for SRI applications.

We demonstrate that fluorescently conjugated mSA is compatible with a wide range of microscopy techniques, including uPAINT, Fluorescence Recovery After Photobleaching (FRAP), STED and dSTORM, in both live and fixed conditions. We further show that, unlike antibodies or streptavidin, mSA labels its targets without crosslinking and penetrates deep into live tissues. We applied mSA-based labelling to probe several synaptogenic adhesion proteins in neurons, including the presynaptic neurexin-1β (Nrx1β), and its postsynaptic binding partners neuroligin-1 (Nlg1), and leucine-rich-repeat transmembrane protein 2 (LRRTM2), against which good antibodies compatible with live cell imaging are lacking. We demonstrate differential nanoscale organization and dynamics of Nlg1 and LRRTM2, which may underlie divergent physiological roles at the synapse.

## Results

### mSA labels biotinylated proteins at the cell surface

Recombinant mSA was produced in bacteria, purified by affinity chromatography via a 10-His N-terminal tag and covalently conjugated to photostable organic dyes Atto 594 or Atto 647N for uPAINT and STED experiments, respectively, or to Alexa 647 for dSTORM imaging, using standard N-hydroxysuccinimide (NHS) ester coupling onto solvent-exposed primary amines (Supplementary Fig. 1). Fluorophore-conjugated mSA was used to label membrane proteins in neurons, COS-7 or HEK-293 cells that incorporate an extracellular AP tag. Biotinylation of the AP tag occurred during protein maturation by co-expressed *Escherichia coli* biotin ligase, BirA^ER^ (ref. 17). When added to the extracellular imaging solution, the mSA–dye conjugate bound specifically to cells co-expressing AP-tagged proteins and BirA^ER^, and not to cells expressing either haemagglutinin (HA)-tagged proteins and BirA^ER^, or AP-tagged proteins but lacking BirA^ER^ (Supplementary Fig. 2a–d). mSA labelling was efficient on all cell types and membrane proteins tested and produced images with high signal-to-noise ratio. The relatively fast dissociation kinetics of mSA (*k*_off_=1.05 × 10^−3 ^s^−1^)^25^,^26^ allow efficient release of bound mSA with excess free biotin. In this regard, addition of 200 μM biotin to the labelling solution resulted in rapid loss of the mSA label (Supplementary Fig. 2e,f). Such reversible binding may be exploited to visualize internalized protein complexes using competition with modified biotin having reduced membrane permeability^27^, and synthesized for this study (Supplementary Fig. 3 and Supplementary Movie 1). Covalent labelling or labelling with high-affinity streptavidin derivatives, for example, monovalent tetramer (*k*_off_ ∼4.2 × 10^−6 ^s^−1^)^17^,^24^,^28^, would not allow such measurements. Taken together, these results demonstrate the high specificity of mSA labelling for recombinant biotinylated proteins in a cellular context.

### mSA reports on protein dynamics without crosslinking

**Figure 1:**
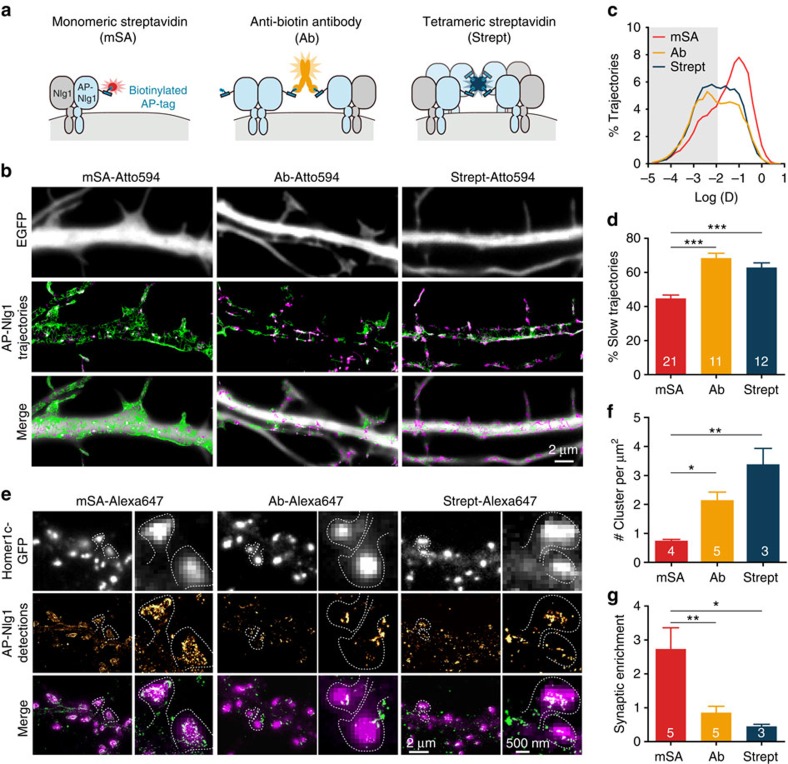
Super-resolution imaging of AP-Nlg1 with mSA or biotin antibody or tetrameric streptavidin. (**a**) Schematic diagram of AP-Nlg1 labelled with three different probes (mSA, monoclonal biotin antibody or streptavidin), conjugated to Atto 594 for uPAINT or Alexa 647 for dSTORM. (**b**) Examples of DIV 7 neurons co-expressing EGFP as a volume marker, AP-Nlg1 and BirA^ER^, and labelled as described above. Middle panels display AP-Nlg1 trajectories calculated from stacks of 4,000 images with 20-ms exposure time (green: fast-diffusing pool, that is, *D*>0.01 μm^2^ s^−1^; magenta: slow diffusing pool, that is, *D*<0.01 μm^2^ s^−1^). Merged images show EGFP (grey) overlaid with AP-Nlg1 trajectories. Note that mSA explores larger surface areas with faster diffusion than multivalent ligands. (**c**) Distribution of AP-Nlg1 diffusion coefficients in a semi-log plot, where the grey-shaded area represents slow trajectories (that is, with *D*<0.01 μm^2^ s^−1^). (**d**) Corresponding percentage of slow trajectories measured in the three different conditions (***P<0.0001). Data are from three different experiments. (**e**) Examples of DIV 15 neurons co-expressing Homer1c-GFP, AP-Nlg1 and BirA^ER^, and labelled with the three Alexa647-conjugated probes shown in **a**. Top panels: Homer1c-GFP signal showing mature synapses (white). Middle panels: super-resolved AP-Nlg1 detection maps generated from 40,000 frames with 20-ms integration time. Bottom panels: merged images showing AP-Nlg1 detections (green) overlaid with Homer1c-GFP (magenta). Note the presence of large AP-Nlg1 aggregates in anti-biotin and streptavidin-labelled neurons. (**f**) Histogram showing the number of Nlg1 clusters per μm^2^ in the three conditions (**P<0.01). (**g**) Histogram showing the synaptic enrichment of AP-Nlg1 compared with the shaft (**P<0.01, *P<0.05). Data are from two different experiments. Numbers in the bar charts represent the number of cells examined.

To evaluate the impact of probe valence on observed protein dynamics, we used uPAINT^8^ to measure the diffusion of biotinylated AP-tagged Nlg1 (AP-Nlg1) in neurons that were labelled with divalent biotin antibody, tetrameric streptavidin or mSA conjugated with Atto 594 (Fig. 1a and Supplementary Fig. 1). The three probes were added at a low concentration (1 nM) to isolate single Nlg1 molecules diffusing on the cell surface. Neurons electroporated with AP-Nlg1 exhibited a twofold stronger surface labelling with Nrx1β-Fc and twofold higher anti-Nlg1 signal in western blots than control neurons expressing empty vector or Homer1c-green fluorescent protein (GFP), indicating a ratio of approximately one overexpressed AP-Nlg1 molecule for one endogenous Nlg1 molecule (Supplementary Fig. 4). In immature neurons, at 7 days *in vitro* (7 DIV), the distribution of AP-Nlg1 diffusion coefficients after mSA labelling was centred around 0.1 μm^2^ s^−1^, revealing fast Brownian motion (Fig. 1b,c). When the movement of AP-Nlg1 was tracked with Atto-conjugated biotin antibody or tetrameric streptavidin, the measured diffusion coefficients were shifted towards lower values (Fig. 1b,c) and there was a concomitant increase in the fraction of slowly diffusing molecules (Fig. 1d), defined as *D*<0.01 μm^2^ s^−1^ (Supplementary Fig. 5). The surface area covered by AP-Nlg1 trajectories was also higher with mSA than with biotin antibody or streptavidin (Fig. 1b). Together, these results suggest that divalent and tetravalent probes alter AP-Nlg1 distribution and dynamics through a combination of protein crosslinking and steric hindrance.

### mSA prevents aggregation compared with multivalent probes

To examine the effects of probe size and valence on Nlg1 distribution at the nanoscale, we used dSTORM to image Nlg1 in mature neurons (DIV 15)^5^,^29^,^30^. Membrane-bound AP-Nlg1 was labelled with 100 nM mSA, biotin antibody or streptavidin conjugated with Alexa 647 for 10 min before fixation and observation. In contrast to our uPAINT experiments, which rely on sparse labelling and short acquisition sequences to focus on protein dynamics, dSTORM uses high-density labelling combined with long acquisition times (800 s) to produce a static representation of the overall protein organization. In parallel, postsynaptic densities were identified using co-expressed Homer1c-GFP^31^,^32^. mSA labelling was fairly diffuse in the shaft and highly concentrated at synapses (Fig. 1e). In contrast, both biotin antibody and streptavidin induced the aggregation of AP-Nlg1 into numerous small clusters on dendrites (Fig. 1e,f), which are most likely caused by ligand-mediated receptor crosslinking. The anti-biotin-labelled AP-Nlg1 was found mostly on the edge of the synapses around rather than inside, presumably because the antibody has limited accessibility to the synaptic cleft because of its large size. With its intermediate size, tetrameric streptavidin penetrated well inside synapses, but because of massive extrasynaptic clustering was less enriched than anti-biotin at synapses compared with shaft levels. Overall, the enrichment of AP-Nlg1 at Homer1c-GFP-positive puncta was significantly higher for mSA compared with streptavidin and antibody (Fig. 1g), indicating that mSA enables a more accurate visualization of receptor localization and organization at the nanometre scale.

### mSA efficiently labels Nlg1 in the confined synaptic cleft

**Figure 2:**
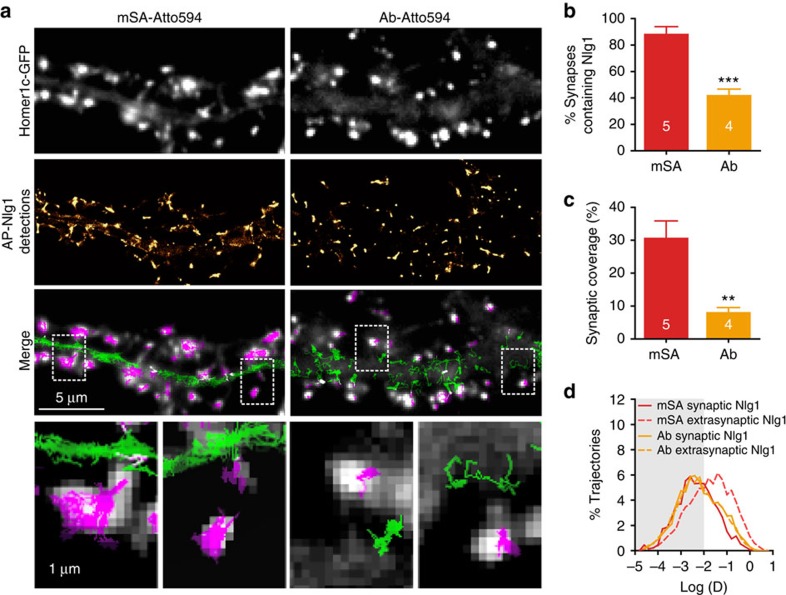
Different ability of mSA and biotin antibody to label AP-Nlg1 within the synaptic cleft in live conditions. (**a**) DIV 15 neurons expressing Homer1c-GFP, AP-Nlg1 and BirA^ER^ were labelled with mSA or anti-biotin conjugated to Atto594 to track individual AP-Nlg1 molecules by uPAINT. From top to bottom: Homer1c-GFP signal staining mature synapses; super-resolved AP-Nlg1 detection maps; merged images showing extrasynaptic AP-Nlg1 trajectories (green) and synaptic trajectories (magenta) overlaid with Homer1c-GFP signals (grey); insets show that mSA-stained Nlg1 fills the entire synaptic area, whereas antibiotin remains on the edge of the postsynaptic density. (**b**) Percentage of synapses containing AP-Nlg1 labelled with mSA or biotin antibody (***P<0.0001). (**c**) Percentage of the synaptic area occupied by AP-Nlg1 when labelled with mSA or anti-biotin (**P<0.01). (**d**) Semi-log distribution of synaptic (solid lines) and extrasynaptic (dashed lines) diffusion coefficients for AP-Nlg1 measured with the two different probes. Data are from two different experiments. Numbers in the bar charts represent the number of cells analysed.

To assess the ability of mSA-labelled Nlg1 to dynamically access the synaptic cleft (20 nm across), we performed uPAINT experiments on live DIV 10–15 neurons expressing AP-Nlg1, and labelled using either mSA or biotin antibody conjugated to Atto594. Using mSA, we observed a gradual decrease in AP-Nlg1 diffusion across neuronal development (Supplementary Fig. 6a–c), revealing the stabilization of Nlg1 associated with an increase in synapse density and maturation^33^. On DIV 15, AP-Nlg1 trajectories recorded with mSA populated both the dendritic shaft and synapses and substantially overlapped with the synaptic Homer1c-GFP staining (Fig. 2a and Supplementary Movie 2), whereas AP-Nlg1 trajectories obtained with biotin antibody were mostly localized on the dendritic shaft and confined to areas around the postsynaptic density. Quantitatively, 85% of synapses contained mSA-labelled Nlg1, while only 40% contained antibody-labelled Nlg1 (Fig. 2b), and the synaptic area covered by mSA-labelled Nlg1 was fourfold higher than antibody-labelled Nlg1 (30% versus 8%, respectively; Fig. 2c). mSA-labelled Nlg1 exhibited fast diffusion in extrasynaptic compartments and slower diffusion at synapses (Fig. 2d), reflecting interactions with presynaptic axonal adhesion protein, Nrx and/or postsynaptic scaffolding proteins^32^. In contrast, anti-biotin labelling showed consistently slower diffusion across the entire neuronal surface, similar to the observations on DIV 7 (Fig. 1c). An additional level of immobilization of mSA-labelled AP-Nlg1 on DIV 15 was observed on incubation with soluble dimeric Nrx1β-Fc (Supplementary Fig. 6d,e), which is consistent with the preferential anchorage of Nlg1 to synaptic PSD-95 triggered by Nrx1β binding^32^. These observations show that mSA can be used to label both fast- and slow-moving AP-Nlg1 in developing neurons, and to visualize trapping events in mature synapses without introducing steric and valence bias compared with divalent antibodies.

### mSA labels target proteins in organotypic brain tissue

**Figure 3:**
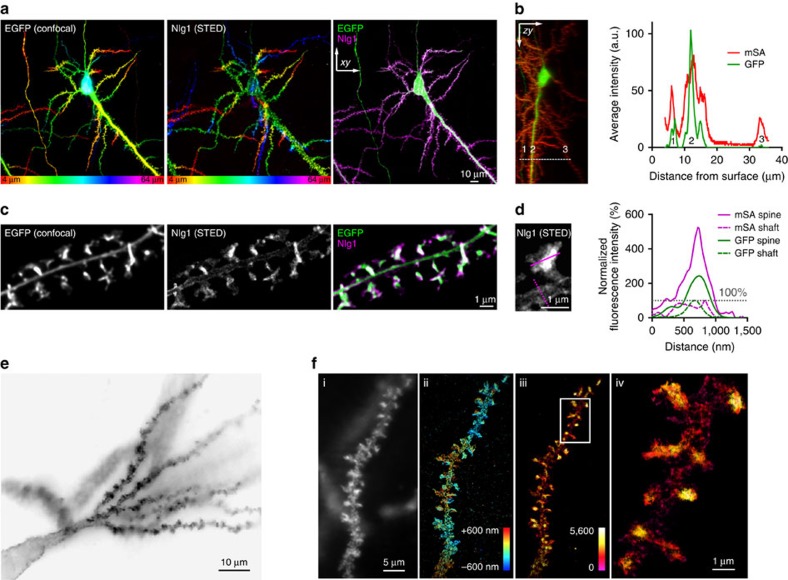
Super-resolution imaging of AP-Nlg1 in organotypic hippocampal slices. (**a**) Confocal (EGFP, left), STED (mSA-Atto 647N, middle) and merged (right) images acquired from live neurons expressing GFP, AP-Nlg1 and BirA^ER^ in organotypic hippocampal slices. Images are projections of a z-stack of 60 planes taken by 1-μm increments and colour-coded with respect to sample depth. (**b**) Linescan measurements of mSA-Atto 647N and GFP staining along the *z* axis. The reduction in mSA-Atto 647N intensity with the sample depth likely reflects reduced laser penetration rather than weaker staining, since the GFP signal also decreases with depth. (**c**) High-magnification deconvoluted STED projection of mSA-labelled Nlg1 in a hippocampal slice shows the accumulation of Nlg1 at dendritic spines. (**d**) Linescans of GFP and mSA-Atto 647N fluorescence intensity in the shaft membrane and in a dendritic spine normalized to the respective fluorescence of GFP and Atto 647N in the shaft. (**e**) Wide field image of mSA-Alexa647 selectively labelling one neuron expressing GFP, AP-Nlg1 and BirA^ER^, a few microns deep from the surface of an organotypic brain slice. (**f**) Astigmatic-based 3D dSTORM imaging in an organotypic brain slice from Nlg1 KO mice: (i) wide field image of mSA-Alexa647-labelled AP-Nlg1. (ii) 3D dSTORM-reconstructed image of a dendritic segment based on 1,038,506 single-molecule localizations from 64,000 images. The image is colour-coded with respect to the *z* distance (−600 to +600 nm). (iii) Normalized localization detection maps integrated within z=±400 nm. (iv) Magnified view of iii showing the enrichment of Nlg1 in dendritic spines.

Given the small size and high labelling efficiency of mSA, we tested its capability to label neurons in dense organotypic tissue. We co-expressed GFP, AP-Nlg1 and BirA^ER^ in individual CA1 pyramidal neurons from 150-μm-thick mouse organotypic hippocampal slices using single-cell electroporation. Slices were labelled by one-step incubation with 100 nM mSA-Atto 647N (for STED) or mSA-Alexa647 (for dSTORM), then rinsed and observed live (STED) or after fixation (dSTORM). The mSA-labelling of AP-Nlg1 was highly specific since it stained only GFP-co-expressing cells throughout the slice with extremely low background, and localized exclusively to somatodendritic regions (Fig. 3a,b and Supplementary Movie 3). The photostability of Atto dyes combined with the thermal stability of mSA allowed an extensive live STED imaging of optical sections at 37 °C down to 40 μm deep. Zoom on dendritic portions showed specific enrichment of Nlg1 in spines compared with GFP (Fig. 3c,d and Supplementary Movie 4), which is also observed in dissociated hippocampal cultures (Fig. 1e–g).

Using conventional confocal microscopy, we evaluated how well mSA penetrates into brain slices compared with streptavidin and biotin antibodies. mSA and tetrameric streptavidin were able to label AP-Nlg1 expressed in live electroporated neurons within a few minutes, although the labelling efficiency of streptavidin decreased rapidly with depth, suggesting an impact of the probe size and/or valence in reaching deep tissues (Supplementary Fig. 7a,b). At the same sample depth (∼15 μm below the surface of the slice), the anti-biotin signal was not detectable after a 10-min live labelling, but became apparent after 1 h (Supplementary Fig. 7c). Finally, we used astigmatism-based three-dimensional (3D) dSTORM to image the distribution of mSA-labelled AP-Nlg1 in small dendritic regions on the surface of a slice from Nlg1 knockout (KO) mice, revealing high enrichment of Nlg1 in spines (Fig. 3e,f and Supplementary Movie 5). These results show that mSA penetrates deep into thick tissues, where it can be used to label target proteins at high density and with specificity for immediate application in SRI. Interestingly, a similar AP-Nlg1 localization was observed in STED images made on organotypic slices from wild-type mice, and in STORM images made on organotypic slices from Nlg1 KO mice, where AP-Nlg1 expressed in CA1 cells replaces endogenous Nlg1.

### mSA and GFP nanobody similarly probe protein diffusion

**Figure 4:**
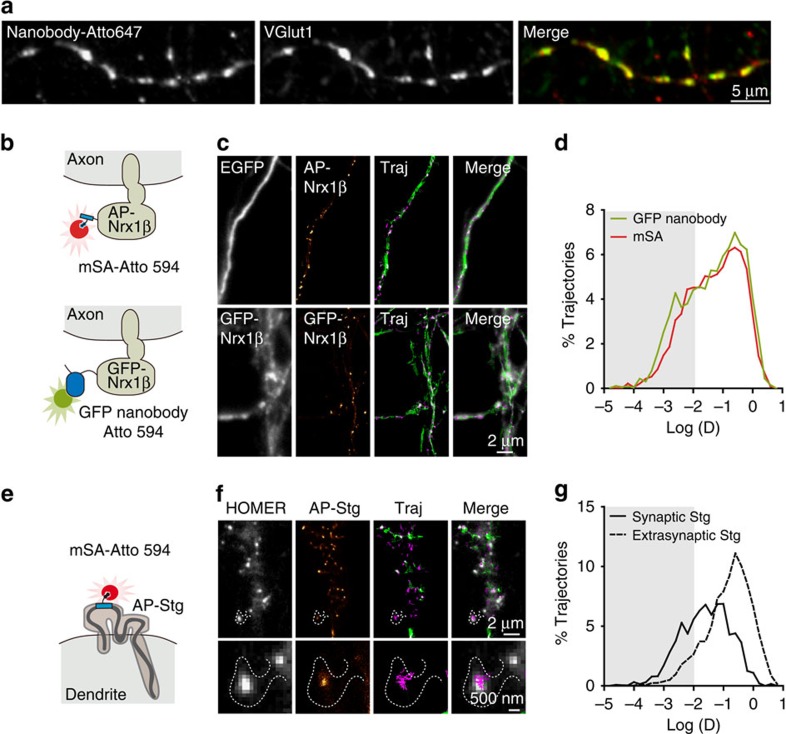
Comparison of mSA and GFP nanobody. (**a**) DIV 15 axons expressing GFP-Nrx1β were live-labelled using anti-GFP nanobody-Atto647, followed by immunolabelling for VGlut1 to stain pre-synaptic glutamatergic terminals. The merged image shows the colocalization between GFP-Nrx1β (red) and VGlut1 (green). (**b**) Schematics showing the labelling of AP-Nrx1β with mSA-Atto594 (top) and GFP-Nrx1β with GFP nanobody-Atto594 (bottom). (**c**) Examples of axonal regions from DIV 15 neurons expressing EGFP, AP-Nrx1β and BirA^ER^ (top); or GFP-Nrx1β (bottom). From left to right: GFP signal, Nrx1 β detection maps, colour-coded Nrx1β trajectory maps (green: fast-diffusing pool, that is, *D*>0.01 μm^2^ s^−1^; magenta: slow diffusing pool, that is, *D*<0.01 μm^2^ s^−1^), merged image showing Nrx1β trajectories overlaid with GFP (grey). (**d**) Distributions of the diffusion coefficients obtained for AP-Nrx1β or GFP-Nrx1β (mSA, *n*=6; GFP nanobody, *n*=5 cells from two different experiments). (**e**) Schematics showing the structure of the AMPA receptor auxiliary protein stargazin (Stg) with the insertion of an AP tag in the first extracellular protein loop. (**f**) Example of DIV 15 neurons co-expressing the synaptic marker Homer1c-GFP, AP-Stg and BirA^ER^. AP-Stg individual molecules were tracked using mSA-Atto594 by uPAINT. Super-resolved AP-Stg localization and trajectory maps were reconstructed from 4,000 images of 20-ms exposure time, with the same colour code as in **b**. (**g**) Diffusion coefficient distribution of AP-Stg inside and outside synapses (*n*=6 cells from two different experiments).

Monomeric VhH antibodies (nanobodies) against GFP and red fluorescent protein have been previously used for SRI, where their small size provides smaller linkage error relatively to the target structure^13^,^14^. We thus sought to compare mSA to these well-characterized tools. To this end, we labelled the same axonal molecular target, Nrx1β, using both approaches and compared the measured diffusion dynamics and membrane organization. On the one hand, we labelled Nrx1β carrying a biotinylated N-terminal AP tag with mSA-Atto594. On the other hand, Nrx1β fused with an N-terminal GFP tag was labelled with Atto594-conjugated GFP nanobody, resulting in a specific enrichment at excitatory pre-synapses immunostained for the vesicular transporter VGlut1 (Fig. 4a). Single-molecule trajectories were then recorded for each labelling scheme using uPAINT in DIV 15 neurons (Fig. 4b,c). The distributions of diffusion coefficients were very similar for the two probes (Fig. 4d), with a major fast diffusion peak centred around ∼0.5 μm^2 ^s^−1^, representing free diffusion in extrasynaptic compartments, and a minor slower diffusion peak around 0.005 μm^2 ^s^−1^, representing Nrx1β confined at presynaptic terminals^34^,^35^. These results show that, in agreement with their small size and monomeric nature, mSA and nanobody labelling lead to similar diffusion measurements. Given their orthogonality, the two approaches may thus be combined for dual-colour SRI.

### The AP tag can be inserted into small extracellular loops

An advantage of using mSA is that the short 15-amino-acid AP tag can be readily incorporated into extracellular protein domains, such as protein loops, without perturbing the native function. To demonstrate the use of the AP tag in a system that may not be amenable for GFP fusion, we inserted the AP sequence in the first extracellular loop of the alpha-amino-3-hydroxy-5-methyl-4-isoxazole-propionic acid (AMPA) receptor auxiliary protein stargazin (AP-Stg)^36^ (Fig. 4e). Using mSA-Atto594, we observed the localization and dynamics of AP-Stg in DIV 15 neurons by uPAINT (Fig. 4f). AP-stargazin localized at synapses where its diffusion was reduced, consistent with previously published work^37^ (Fig. 4f,g). Thus, the mSA-labelling strategy can be successfully applied to situations where the use of a GFP tag may not be allowed for structural or functional reasons. To further illustrate this point, we inserted the mCherry FP (256 amino acids) in the extracellular loop of stargazin in place of the AP tag (15 amino acids). When expressed in heterologous COS-7 cells, this fusion protein formed numerous vesicular aggregates enriched in a perinuclear area, suggesting that mCherry-stg is retained in intracellular transport compartments, and not properly expressed at the cell membrane, possibly because of protein misfolding (Supplementary Fig. 8). In contrast, fusing mCherry to the C terminus of stargazin resulted in very clear membrane localization. This experiment clearly shows that AP tags can be advantageous compared with large domains such as FPs for the tagging of complex membrane proteins.

### Dual-colour imaging of *trans*-synaptic adhesive contacts

**Figure 5:**
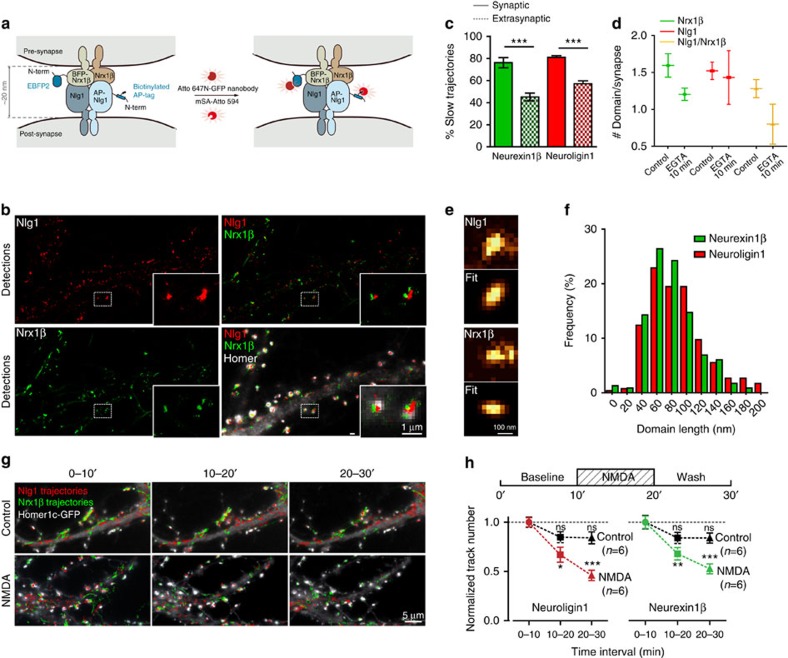
Dual-colour super-resolution imaging of *trans*-synaptic contacts between neurexin-1β and neuroligin-1. Neurons co-expressing AP-Nlg1, BirA^ER^ and Homer1c-GFP or expressing BFP-Nrx1β were co-cultured for 15 days and labelled with mSA-Atto 594 and Atto 647N nanobody. (**a**) Schematics of labelled adhesion molecules at the synapse. (**b**) Integrated density of Nlg1 (red) and Nrx1β (green) molecules at axon/dendrite contacts, identified from Homer1c-GFP signal (grey). (**c**) Percentages of slow trajectories for Nlg1 (red) and Nrx1β (green) were measured inside (solid) and outside (stippled) synapses (Nrx1β, *n*=12; Nlg1, *n*=10 cells from three different experiments ***P<0.0001). (**d**) Number of Nrx1β (green), Nlg1 (red) and apposed Nrx1β/Nlg1 (yellow) synaptic clusters before and after 10 min EGTA treatment (*n*=6 cells for each condition from two different experiments). (**e**) Examples of synaptic Nlg1 and Nrx1β clusters and corresponding two-dimensional anisotropic Gaussian fits. (**f**) Frequency distributions of Nlg1 and Nrx1β cluster lengths (Nlg1: median 80.44, interquartile range (IQR) 56–102 nm, 531 clusters from 14 cells; Nrx1β: median 75.17, IQR of 62–109 nm, 235 clusters from 11 cells. Data are from three different experiments). (**g**) Destabilization of *trans*-synaptic Nrx1β/Nlg1 contacts by NMDA application (20 μM, 10 min). (**h**) Number of Nlg1 and Nrx1β trajectories over time on a 10-min NMDA treatment (red, green) or in control condition (black). The trajectory counts were normalized by their respective numbers before treatment (*n*=6 cells for each condition from three different experiments ***P<0.0001, one-way analysis of variance).

Postsynaptic Nlg1 interacts with presynaptic Nrx1β to form *trans*-synaptic contacts^38^. To visualize the dynamic organization of mature Nrx1β/Nlg1 complexes, we used mSA in combination with GFP nanobody^13^,^14^ for dual-colour uPAINT analysis. Neurons expressing either AP-Nlg1 or a blue fluorescent protein Nrx1b fusion (BFP-Nrx1β) were co-cultured for 2 weeks, and the individual AP-Nlg1 and BFP-Nrx1β molecules on the surface of dendrites and axons, respectively, were labelled with mSA-Atto 594 and GFP nanobody-Atto 647N for tracking (Fig. 5a,b and Supplementary Movie 6). Both BFP-Nrx1β and AP-Nlg1 showed decreased diffusion at Homer1c-GFP-positive areas, resulting in an increase in the slowly moving fraction (Fig. 5b,c), which is consistent with the formation of functional *trans*-synaptic complexes. The images reconstructed from all single-molecule detections indicate that every synapse contains one to two domains of Nrx1β and Nlg1 hot spots facing each other (Fig. 5d), with an average diameter of 85±3 nm for Nrx1β and 91±2 nm for Nlg1 (Fig. 5e,f). Given the dilute labelling conditions, these domains reflect the trapping of a small number of molecules at the synapse, whose intensity compared with diffusive molecules is amplified by the temporal integration of long-emitting fluorescent signals coming from the same location. Indeed, the domain size is in agreement with the confinement diameter measured from the mean squared displacement of synaptic Nlg1 molecules (Supplementary Fig. 5).

Addition of ethylene glycol tetra-acetic acid (EGTA) as a calcium chelator decreased the number of synaptic Nrx1β and Nlg1 confinement domains (Fig. 5d) and increased the diffusion of both proteins (Supplementary Fig. 9). Both changes are likely caused by a dissociation of calcium-dependent Nrx1β/Nlg1 bonds^39^, followed by lateral motion of the two proteins. We also used dual-colour uPAINT to examine the dynamics of Nrx1β/Nlg1 adhesion in response to synaptic stimulation in live conditions. We treated neurons with 20 μM N-methyl-D-aspartate (NMDA) for 10 min to chemically induce synaptic depression^40^ and monitored Nrx1β and Nlg1 dynamics by uPAINT. NMDA caused a specific and time-dependent decrease in BFP-Nrx1β and AP-Nlg1 molecules from the cell surface (Fig. 5g,h), whereas their number remained constant under control conditions. Such a loss of the Nrx1β/Nlg1 complex thus represents coordinated, activity-dependent destabilization of *trans*-synaptic contacts at the single-molecule level.

### Differential dynamics of Nlg1 and LRRTM2 at synapses

**Figure 6:**
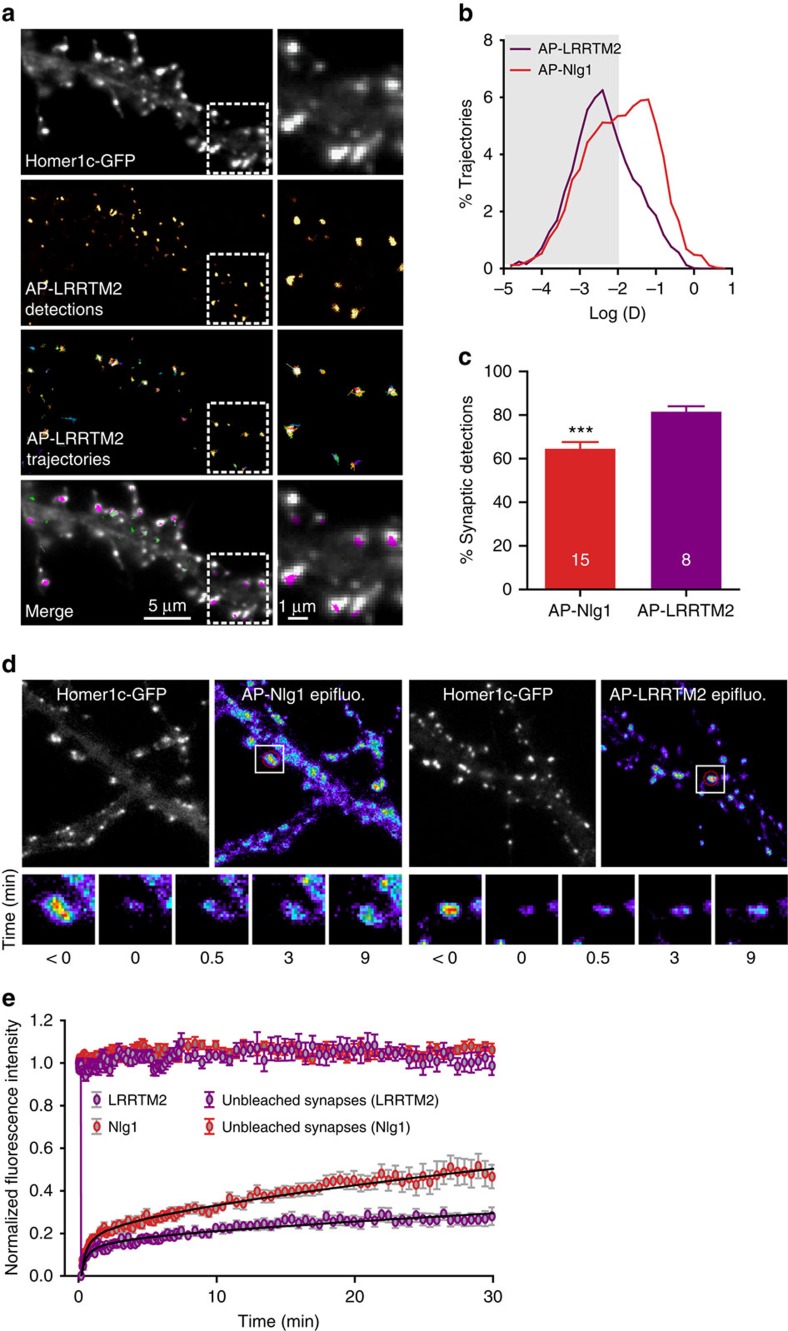
Comparison of Nlg1 and LRRTM2 dynamics in mature hippocampal neurons. (**a**) DIV 15 neurons expressing AP-LRRTM2, Homer1c-GFP and BirA^ER^ were labelled using mSA-Atto 594 for uPAINT imaging of single LRRTM2 molecules. Super-resolved localization and trajectory maps are shown (green, fast-diffusing molecules, magenta, slow-moving molecules). Note the absence of diffusion on dendritic shafts. (**b**) Semi-log distribution of AP-LRRTM2 and AP-Nlg1 diffusion coefficients. (**c**) Corresponding percentage of synaptic LRRTM2 and Nlg1 detections by uPAINT (LRRTM2, *n*=8; Nlg1, *n*=15 cells from three different experiments). (**d**) FRAP experiments performed on AP-Nlg1 and AP-LRRTM2 labelled with mSA-Atto594. (**e**) Corresponding normalized fluorescence recovery curves. The intensity of unbleached synapses is shown as control for observational photobleaching. Solid lines represent fits of the mean data points with the diffusion-reaction equation given in the Methods. The parameters obtained for Nlg1 and LRRTM2 were the fraction of free molecules *φ*=0.27 and 0.19 and the turnover rate of adhesions *k*_reac_=1.4 × 10^−2^ and 5.0 × 10^−3 ^min^−1^, respectively. The ratio of all synaptic molecules versus free molecules (1/*φ*) gives 3.5 for Nlg1 and 5.1 for LRRTM2, closely corresponding to the synaptic enrichment values measured by dSTORM (Nlg1, *n*=25; LRRTM2, *n*=18; Nlg1 unbleached, *n*=10; LRRTM2 unbleached, *n*=8 cells for each condition from three different experiments).

To gain insight into the membrane dynamics of other synaptogenic proteins, we examined the behaviour of another excitatory postsynaptic adhesion molecule, LRRTM2, that competes with Nlg1 for the binding to presynaptic neurexins^41^,^42^,^43^. We first used the same dual-colour uPAINT analysis described above to simultaneously image AP-LRRTM2 in dendrites and BFP-Nrx1β in axons. Similarly to Nlg1, LRRTM2 was highly concentrated at synapses, where it formed a small number of confinement domains of 118±4 nm diameter overlapping with BFP-Nrx1β (Supplementary Fig. 10). However, the average diffusion coefficient of LRRTM2 was significantly lower than that of Nlg1, reflecting the fact that most LRRTM2 molecules (80%) were localized at synapses, while very few (20%) diffused freely on dendritic shafts (Fig. 6a–c). For comparison, the synaptic and dendritic fractions of Nlg1 were 60% and 40%, respectively.

To confirm that Nlg1 and LRRTM2 also exhibited differential dynamics at the population level, we performed FRAP experiments. We labelled AP-Nlg1 and AP-LRRTM2 at the cell surface with 100 nM mSA-Atto594 and photobleached individual synapses using a focused 561-nm laser beam. Fluorescence recovery was analysed for up to 30 min (Fig. 6d,e). Control FRAP experiments on Nlg1-GFP showed similar recovery curves (Supplementary Fig. 11). The recovery curves were fitted with a diffusion-reaction model described previously, which includes three adjustable parameters: the fraction of adhesion molecules trapped at the synapse, their steady-state exchange rate and the diffusion coefficient of free molecules^44^. We used the fastest AP-Nlg1 and LRRTM2 observed in uPAINT to estimate the diffusion coefficient of free molecules (∼0.1 μm^2 ^s^−1^) and adjusted the two other parameters to obtain the best fit. The exchange rate was approximately threefold lower for AP-LRRTM2 than for AP-Nlg1, and the trapped fraction was higher (81% versus 73%, respectively). Therefore, FRAP predicts a longer synaptic residence time for LRRTM2 than for Nlg1, consistent with the uPAINT data.

### Differential nanoscale organization of Nlg1 and LRRTM2

**Figure 7:**
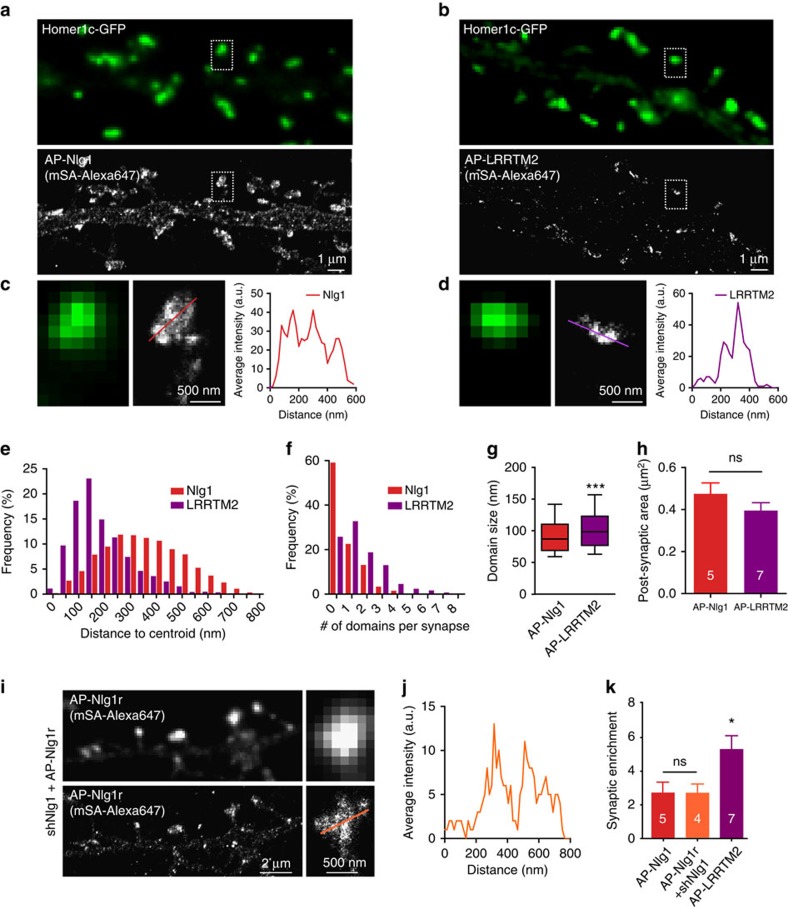
Comparison of Nlg1 and LRRTM2 organization in mature hippocampal neurons. (**a**,**b**) DIV 15 neurons expressing either AP-Nlg1 or AP-LRRTM2, plus Homer1c-GFP and BirA^ER^ were labelled at high density with mSA-Alexa 647 for dSTORM imaging. Integrated densities over 40,000 frames are shown. (**c**,**d**) AP-Nlg1 and LRRTM2 fluorescence across linescans represented in **a,b** insets show local fluorescence accumulation within synapses. (**e**) Dispersion of AP-Nlg1 and AP-LRRTM2 molecules within synapses represented as the distribution of distances from individual synaptic detections relatively to the centroid of the Homer1c-GFP signal (LRRTM2, *n*=7; Nlg1, *n*=5). (**f**) Distribution of the number of locally enriched AP-Nlg1 and AP-LRRTM2 domains within synapses (LRRTM2, *n*=7; Nlg1, *n*=5). (**g**) The median sizes of AP-Nlg1 and AP-LRRTM2 locally enriched synaptic domains (Nlg1, 87.00, IQR 68–110, *n*=5; LRRTM2, 98.35, IQR 76–122, *n*=7; ***P<0.0001). (**h**) Synaptic area in DIV 15 neurons electroporated with AP-Nlg1 or AP-LRRTM2 based on the Homer1c-GFP signal. (**i**) Representative STORM image of AP-Nlg1r expressed on a knockdown background in DIV 15 rat neurons, shown with the corresponding low-resolution mSA-Atto647 labelling. (**j**) Average intensity corresponding to the linescan in **i** showing local AP-Nlg1r fluorescence accumulation within a spine in a knockdown background, similar to AP-Nlg1. (**k**) Synaptic enrichment of AP-Nlg1, AP-Nlg1r co-expressed on a knockdown background and AP-LRRTM2 with respect to shaft levels (AP-Nlg1, 2.73±0.62, *n*=5; AP-Nlg1r, 2.54±0.34, *n*=4; AP-LRRTM2, 5.29±0.81 *n*=7; *P<0.05). Data are from three different experiments for AP-Nlg1 and AP-LRRTM2, and two experiments for AP-Nlg1r.

Finally, to gain insight on the nanoscale organization of Nlg1 and LRRTM2, we imaged Nlg1 and LRRTM2 by dSTORM in mature neurons (Fig. 7a–d). Unlike in uPAINT, originally diffusing molecules appear with a similar intensity to confined ones because of fixation, thus contributing to a homogenous signal in the shaft and spines. This effect was especially prominent for Nlg1, which was more diffusive than LRRTM2, resulting in a lower synaptic enrichment when compared with shaft levels (2.7±0.6 versus 5.3±0.8, respectively). Furthermore, the dense labelling allows the identification of many simultaneous Nlg1-trapping events in the dendritic spine, yielding a fairly disperse localization with respect to the synapse centroid (Fig. 7e), but occasionally forming one or two clusters of similar sizes as those observed with uPAINT (average diameter 98±6 nm; Fig. 7f,g). In contrast, LRRTM2 seemed to visit a more restricted postsynaptic area, mostly forming one compact mass comprising several small subclusters (107±7 nm) and being localized at a shorter distance from the centroid (Fig. 7b,d–g). This specific scattering of Nlg1 was not due to an increase in postsynaptic size compared with LRRTM2 (Fig. 7h). In summary, the two Nrx partners, Nlg1 and LRRTM2, differed considerably in their postsynaptic nano-organization and dynamics.

Finally, to rule out the possibility of a localization artefact due to AP-Nlg1 overexpression, saturating synaptic binding sites, we designed a rescue strategy by replacing endogenous Nlg1 with similar levels of exogenous AP-Nlg1. To this aim, we either co-expressed a short hairpin RNA to Nlg1 together with a resistant AP-Nlg1 construct in rat cultures, or expressed AP-Nlg1 in primary hippocampal cultures from Nlg1 KO mice. Western blots and Nrx1β-binding assays confirmed that exogenous AP-Nlg1 levels roughly reached those of endogenous Nlg1 in both approaches (Supplementary Fig. 4). STORM images performed with mSA-Alexa647 on these cultures revealed a very similar distribution of rescue AP-Nlg1 compared with what we had previously observed using AP-Nlg1 expression alone, with a similar spine/shaft enrichment (Fig. 7i–k). Together with the STORM images obtained in organotypic hippocampal slices from Nlg1 KO mice (Fig. 3e,f), these results demonstrate that the relative scattering of AP-Nlg1 within spines most likely reflects the localization of endogenous Nlg1.

## Discussion

In this study, we developed and extensively characterized a new labelling method compatible with SRI, based on fluorescent monomeric streptavidin (mSA), to target recombinant biotinylated proteins at the cell membrane. mSA can be produced using standard bacterial protein production, and conjugated to fluorophores of interest with established coupling protocols. This enables customization of the labelling toolbox with addition of new fluorophores, such as the recently reported silicon–rhodamine derivatives that have improved photostability and fluorogenic properties. The high thermodynamic and aqueous stability of mSA allows for its conjugation to a variety of organic dyes with different physical properties (Atto-565, -594, -647N and Alexa 647) without having an impact on its behaviour in cellular environments, even when some dyes, for example, Atto 647N, are prone to incorporation in the membrane^45^. Although mSA cannot be used to label endogenous proteins, the low abundance of naturally biotinylated proteins^46^ allowed highly specific labelling of target proteins with all mSA conjugates tested. Therefore, using a similar labelling scheme, we were able to explore real-time single-molecule dynamics at the nanoscale level (uPAINT), and to map representative protein organization at a precise time point to measure local densities within immobile structures (STED and dSTORM).

Antibodies are commonly used for labelling; however, high-quality antibodies compatible with live imaging are not always available, a critical issue for the synaptic adhesion molecules studied here. Furthermore, our comparison of monomeric (mSA), dimeric (antibody) and tetrameric (streptavidin) probes clearly shows that, for live SRI applications, probe multivalence affects the measurements by generating artificial nanoscale clusters and biasing protein diffusion. For single-particle-tracking applications, mSA-Atto conjugates represent a significant improvement over large and multivalent nanoparticles such as antibody- or streptavidin-coated quantum dots, which often get sequestered at the periphery of the postsynaptic density because of steric hindrance^31^,^32^,^47^. However, because of their resistance to photobleaching compared with organic fluorophores, nanoparticles will remain a method of choice to track molecules for long durations, even more so with the recently developed non-blinking fluorescent nanoparticles^48^,^49^. mSA labelling is in many regards comparable to that of anti-GFP nanobody labelling, an example of monovalent detection recently used in SRI^13^. In some cases, however, labelling with mSA may be advantageous if the use of GFP as an antigenic tag is problematic. Indeed, the 15-amino-acid AP tag, which is similar in size to other commonly used epitopes, such as FLAG or c-Myc, can be readily inserted between structural domains or within protein loops with minimal perturbation of the native function. We demonstrated this point by fusing the AP tag on a short extracellular loop of stargazin that was previously targeted with an engineered HA tag^36^, whereas FP insertion at this location resulted in a dramatic mislocalization of the resulting fusion construct.

A benefit of developing an orthogonal technique of comparable capability includes the potential for dual-colour SRI. To this end, we simultaneously applied mSA and GFP nanobody labelling of *trans*-synaptic contacts in hippocampal neurons to reveal a differential dynamic organization of the two main Nrx1β adhesion partners at excitatory synapses, namely Nlg1 and LRRTM2. We showed a diffusional trapping of presynaptic Nrx1β and postsynaptic Nlg1, resulting in selective *trans*-synaptic apposition of those proteins at axon–dendrite contacts. Labelling of Nrx1β/Nlg1 complexes was rapid with both mSA and GFP nanobody, neither of which affected their intrinsic interaction. In contrast, some labelling strategies, such as GFP complementation, have intrinsically slower kinetics owing to GFP folding and can artificially increase the binding strength between Nrx and Nlg, thus enhancing synaptogenesis^38^. That Nrx1β escaped synapses more readily than Nlg1 on EGTA treatment suggests that the molecule may be retained at pre-synapses through extracellular calcium-dependent interactions, for example, with Nlgs and LRRTMs. This observation is consistent with the report that the Nrx intracellular domain is dispensable for Nlg1-induced presynaptic differentiation^50^. Nlg1 may be anchored at the synapses not only via extracellular interactions with Nrxs but also via intracellular interactions with PSD-95, which may be promoted by Nrx1β binding^32^.

Whether adhesion molecules are functionally regulated by synaptic activity remains an important, unresolved issue. Nlg1 interacts directly with the extracellular domain of NMDA receptors^51^ and Nlg1 knockdown affects NMDAR-mediated synaptic transmission^52^,^53^. In addition, the synaptogenic effect induced by Nlg1 expression is abolished by chronic blockade of NMDA receptor activity^53^, whereas synapse elimination caused by the absence of Nlgs and LRRTMs is promoted by calcium-calmodulin kinase II (CamKII)-dependent synaptic activity^54^. Our results show that both Nlg1 and Nrx1β *trans*-synaptic clusters rapidly vanish on NMDA treatment, suggesting that Nlg1 plays a role in activity-dependent synaptic remodelling through NMDA receptor activity. Proteolytic shedding of Nlg1 at the cell membrane by the metalloprotease MMP9 (ref. 55) or altered export of Nlg1 through phosphorylation by CamKII (ref. 56) provide possible mechanisms. In contrast to Nlg1, LRRTM2 is less mobile and is confined in more compact synaptic domains, suggesting that the molecule plays a strong role in maintaining *trans*-synaptic connectivity. This specific function of LRRTM2 might be achieved through direct extracellular interactions with presynaptic Nrxs or with postsynaptic AMPA receptors^43^, the latter playing a retrograde role on presynaptic differentiation^57^. Indeed, alternative splicing of presynaptic Nrx3 decreases both postsynaptic LRRTM2 and AMPA receptor levels^58^, and LRRTM knockdown alters AMPA receptor-mediated synaptic transmission and plasticity^52^. Both the number and size of synaptic LRRTM2 clusters match the recently identified AMPA receptor nanodomains^59^, suggesting that they may be structurally and/or functionally related. One interesting mechanism could be that extrasynaptic surface-diffusing AMPA receptors get dynamically trapped at Nrx1β/Nlg1 adhesions through PSD-95 scaffolds^31^,^33^, and then become stabilized at synapses by more durable interactions with LRRTM2.

Obtaining a more integrated view of the molecular ultrastructure of the synapse requires multicolour SRI of adhesion molecules, scaffolding proteins and neurotransmitter receptors. The penetrability and labelling efficiency reached with mSA, together with targeted expression of adhesion proteins such as Nrx and Nlg in intact organisms^60^ and improved optical detection in deep tissue^61^, should enable further investigation of activity-dependent modulation of synaptic protein organization with high spatial and temporal resolution. In addition to addressing specific questions in neuroscience, the mSA-based labelling strategy constitutes a significant progress towards developing a robust staining technique for fluorescence-based super-resolution microscopy, which should stimulate its application to a wide range of questions in fundamental biology.

## Methods

### DNA plasmids

The AP-Nlg1, AP-Nrx1β, pDisplay-HA-6His-AP-CFP and BirA^ER^ constructs^21^,^23^ were kind gifts from A. Ting (MIT, Boston). HA-Nlg1 and short hairpin RNA to Nlg1 (shNlg1) were obtained from P. Scheiffele (Biozentrum, Basel). The AP-Nlg1 rescue was generated by changing the nucleotide sequence of AP-Nlg1 recognized by the shNlg1 5′- gaaggtactggaaatctg -3′ to 5′- gaGggCacGggTaaCTtg -3′, using silent mutations. Myc-LRRTM2 (ref. 43) was a gift from J. de Wit (Leuwen, Belgium). Nlg1 with GFP insertion at position 728aa (Nlg1-GFP) was generously provided by T. Dresbach (Goettingen, Germany)^62^. AP-LRRTM2 was generated using the In-Fusion HD Cloning kit (Clontech), replacing the myc-tag from myc-LRRTM2 by the AP Tag (amino-acid sequence GLNDIFEAQKIEWHE). The AP tag sequence was amplified from pDisplay-HA-6His-AP-CFP. Oligonucleotides used were as follows: LRRTM2-1F, 5′- ACTAGTTGTCCACCCAAATG -3′; LRRTM2-2R, 5′- GCTAGCCGCCATACCCAG -3′; AP-5F, 5′- GGTATGGCGGCTAGCggcctgaacgatatcttcg -3′; AP-6R, 5′- GGGTGGACAACTAGTctcgtgccactcgatctt -3′. Homer1cGFP was a gift from S. Okabe (Tokyo University, Japan). BFP-Nrx1β was derived from human GFP-Nrx1β (gift from M. Missler, Münster University, Germany) by replacing the GFP sequence by PCR-amplified-EBFP2 from pEBFP2-Nuc (obtained from Addgene, plasmid 14893). AP-SEP-transferrin receptor was a kind gift from D. Perrais (IINS Bordeaux). HA-stargazin^36^ was a gift from R. Nicoll (UCSF, USA). AP-stargazin was obtained by inserting a synthetic fragment containing the AP tag between residues 50 and 51 (in the first extracellular loop, same position as HA-stargazin) using HindIII and BspEI restriction sites. For comparison of the impact of FP insertion, mCherry was inserted into the same stargazin construct either at the same position as the AP tag (first extracellular loop, between residues 50 and 51, AgeI and NheI restriction sites) or at position −56 (BglII site) with respect to the C terminus (cytoplasmic tail). The plasmid for bacterial expression of the anti-GFP nanobody^14^ was a kind gift from Alexis Gautreau (LEBS, Gif-sur-Yvette, France). mSA was subcloned from the previously described pRSET-A vector^25^,^26^ into pET-IG, a homemade vector derived from pET-24 (Novagen) and engineered to incorporate after the start codon a decahistidine tag immediately followed by a Tobacco Etch Virus cleavage site (-ENLYFQS-) and no tag on the C terminus.

### Protein expression and purification

Nrx1β-Fc was obtained as follows. Conditioned medium from a stable hygromycin-resistant HEK cell line producing Nrx1β-Fc lacking splice insert 4 was collected, and recombinant Nrx1β-Fc was purified on a protein G column (HiTrap Protein G HP, GE Healthcare) to a concentration of 0.6–1.0 mg ml^−1^ (refs 31, 32). mSA was produced similarly to previously reported methods^25^,^26^, with slight modifications. Briefly, pET-IG-mSA was transformed into E. Coli BL21 codon plus (DE3)-RIL and expressed for 12 h at 16 °C in 300 ml using an autoinduction protocol^63^. The cells were harvested and resuspended in 9% NaCl, transferred into a conical tube and kept at −80 °C until purification. The cell pellet was thawed and resuspended in 40 ml of freshly prepared denaturing lysis buffer (50 mM Tris acetate, pH 8.0, 8 M urea and 40 μl Protease Cocktail III from Calbiochem) and gently mixed for 30 min at 4 °C. Cells were lysed by sonication and lysates were cleared by centrifugation at 10,000*g* (60 min, 4 °C). HIS-Buster Cobalt Affinity gel (AMOCOL, 1 ml) was added and incubated with the supernatant for 2 h at 4 °C. The resin was collected, washed extensively with washing buffer (50 mM TrisOAc, pH 8.0, 300 mM NaCl, 5 mM imidazole and 8 M urea, freshly prepared) and the protein was eluted with 3 × 1 ml of elution buffer (50 mM TrisOAc, pH 8.0, 300 mM NaCl, 500 mM imidazole, 8 M urea, freshly prepared). The elution fractions were added drop by drop to 40 ml of ice-cold PBS buffer (5 mM Na_2_HPO_4_, 5 mM NaH_2_PO_4_ and 150 mM NaCl) containing 0.3 mg ml^−1^ d-biotin, 0.2 mg ml^−1^ oxidized glutathione and 1 mg ml^−1^ reduced glutathione under rapid stirring to refold the protein. The precipitates were removed by centrifugation. The refolded protein was concentrated to ∼1 mg ml^−1^ using Amicon Ultra centrifugal filters with a 10-kDa cutoff. The anti-GFP nanobody was expressed in the same conditions as mSA and purified under native conditions by affinity chromatography using the HIS-Buster Nickel Affinity gel. The protein was dialysed in PBS buffer and concentrated to ∼1 mg ml^−1^ using Amicon Ultra centrifugal filters with a 10-kDa cutoff. mSA and anti-GFP nanobody were kept at 4 °C and used for coupling to fluorophores within 24 h. The modified, negatively charged biotin (Biot-DDDY-COOH) to reduce membrane permeability^27^ was obtained by manual standard Fmoc-based solid-phase peptide synthesis.

### Protein and antibody coupling to fluorophores

All proteins (mSA, anti-GFP nanobody, streptavidin (85878, Sigma-Aldrich), mouse monoclonal anti-biotin (03-3700, Invitrogen)) were prepared in PBS at ∼1 mg ml^−1^. Coupling to Atto 647N, Atto 594 and Alexa 647 was performed following the recommended procedures from the manufacturers (ATTO-TEC and Life Technologies) with the corresponding NHS ester derivatives of each dye. Briefly, labelling was conducted in the dark at room temperature for 1 h. Excess dye was removed using Sephadex G-25 medium (PD MiniTrap G-25, GE Healthcare) by elution with PBS. mSA-Atto 647N, mSA-Atto 594, mSA-Alexa 647 and the Atto 647N-GFP nanobody were further purified to homogeneity by size exclusion chromatography with a Superdex 75 HiLoad 16/60 column (GE Healthcare) on an AKTA purifier system (GE Healthcare) using PBS as a running buffer. Labelled proteins were concentrated to ∼0.2 mg ml^−1^ using Amicon Ultra centrifugal filters with a 10-kDa cutoff. All proteins were aliquoted and flash-frozen for storage at −80 °C until use.

### Cell culture and electroporation

Gestant rat females were purchased weekly (Janvier Labs, Saint-Berthevin, France), while wild-type and Nlg1 KO mouse strains obtained from F. Varoqueaux and N. Brose (MPI Goettingen) were raised in our animal facility. Animals were handled and killed according to European ethical rules. Dissociated hippocampal neurons from E18 Sprague–Dawley rat embryos or P0 mice (from Nlg1 WT or KO background) were prepared as described^64^, and electroporated with the Amaxa system (Lonza) using 500,000 cells per cuvette. The following plasmid combinations were used: (GFP or Homer1c-GFP) + BirA^ER^ + (AP-Nlg1, AP-LRRTM2, AP-STG or AP-Nrx1β) (1.5:1.5:1.5 μg DNA), BFP-Nrx1β or GFP-Nrx1β (4.5 μg DNA), Nlg1-GFP (4 μg DNA). Electroporated neurons were resuspended in Minimal Essential Medium supplemented with 10% horse serum (MEM-HS) and plated on 18-mm coverslips previously coated with 1 mg ml^−1^ polylysine for 2 h at a concentration of 50,000 cells per coverslip. Three hours after plating, coverslips were flipped onto 60-mm dishes containing a glial cell layer in Neurobasal medium (NB for rat cultures or NB-A for mouse cultures) supplemented with 2 mM L-glutamine and 1 × NeuroCult SM1 Neuronal supplement (STEMCELL Technologies) and cultured for 2 weeks at 37 °C and 5% CO_2_. Astrocyte feeder layers were prepared from the same embryos, plated between 20,000 and 40,000 cells per 60-mm dish and cultured in MEM (Fisher Scientific, cat. no. 21090-022) containing 4.5 g l^−1^ Glucose, 2 mM L-glutamine and 10% horse serum (Invitrogen) for 14 days. For biochemistry experiments, electroporated neurons from rats or mice were seeded in a six-well plate coated with 1 mg ml^−1^ polylysine for 2 h at a concentration of 500,000 cells per well. Three hours after plating, the medium was replaced by conditioned Neurobasal medium (rat) or Neurobasal-A medium (mice), supplemented with 2 mM L-glutamine and 1 × NeuroCult SM1 Neuronal supplement (STEMCELL Technologies) and renewed every 3–4 days. Ara-C (3.4 μM) was added at DIV 3 and DIV 13. COS-7 and HEK-293 cells were cultured in DMEM (GIBCO/BRL) supplemented with 10% fetal bovine serum, 100 units ml^−1^ penicillin and 100 μg ml^−1^ streptomycin. Heterologous cells were electroporated with TfR-SEP-AP+BirA^ER^ (1.5:2 μg DNA for 2 million cells) or stargazin-mcherry (3.5 μg DNA for 2 million cells) constructs with the Amaxa system (Lonza) using 500,000 cells per cuvette.

### Neuronal lysates

DIV 14 mouse or rat neuronal cultures were rinsed in ice-cold PBS, and then scraped into 100 μl RIPA buffer (50 mM HEPES, 10 mM EDTA, 0.1% sodium dodecyl sulfate, 1% IGEPAL CA-630 and 0.5% sodium deoxycholate, pH=7.2). Homogenates were kept 15 min on ice and centrifuged at 8,000*g* for 15 min at 4 °C to remove cell debris. Ten microlitres per condition were loaded for western blots.

### SDS–PAGE and immunoblotting

Samples were separated by TGX stain-free precast gels (4-15% gradient, Bio-Rad), ultraviolet-activated with ChemiDoc Touch system (Bio-Rad) for direct imaging of total proteins and then transferred to nitrocellulose membranes for immunoblotting analysis. After blocking with 5% non-fat dried milk in Tris-buffered saline Tween-20 (TBST; 28 mM Tris, 136.7 mM NaCl, 0.05% Tween-20, pH 7.4) for 45 min at room temperature, membranes were incubated with rabbit anti-Nlg1 (129013, Synaptic systems) diluted at 1:1,000 with 0.5% non-fat dried milk in TBST, followed by horse radish peroxidase-conjugated anti-rabbit antibody (Jackson ImmunoResearch) for 1 h at room temperature. Target proteins were detected by chemiluminescence with Super signal West Dura (Pierce) on the ChemiDoc Touch system (Bio-Rad). The theoretical molecular weight of Nlg1 is 93 kDa, but the apparent molecular weight in immunoblots is ∼130 kDa, likely due to glycosylations^65^. For quantification, the intensity of the chemiluminescence signal of each lane was normalized by the total protein signal on the same lane, revealed by the stain-free technology.

### Organotypic cultures and single-cell electroporation

Organotypic hippocampal slice cultures were prepared from either wild-type or Nlg1 KO mice (C57Bl6/J strain), as described below^66^. Briefly, animals at postnatal day 4–6 were quickly decapitated and their brains placed in ice-cold Gey's balanced salt solution under sterile conditions. Hippocampi were dissected out and coronal slices (350 μm) were cut using a tissue chopper (McIlwain) and incubated with serum-containing medium on Millicell culture inserts (CM, Millipore). The medium was replaced every 2–3 days. After 3–4 days in culture, CA1 pyramidal cells were processed for single-cell electroporation with plasmids encoding enhanced GFP (EGFP) along with AP-Nlg1 and BirA^ER^ in equal proportions. The pipette containing 33 ng μl^−1^ total DNA was placed close to the soma of individual CA1 pyramidal neurons. Electroporation was performed by applying three square pulses of negative voltage (10 V, 20 ms duration) at 1 Hz, and then the pipette was gently removed. Three to five neurons were electroporated per slice, and the slice was placed back in the incubator for several days before imaging.

### Single Molecule Tracking (uPAINT)

uPAINT experiments were carried out as previously reported^8^. Cells were mounted in Tyrode solution (15 mM D-glucose, 108 mM NaCl, 5 mM KCl, 2 mM MgCl_2_, 2 mM CaCl_2_ and 25 mM HEPES, pH 7.4) containing 1% globulin-free BSA (Sigma) in an open Inox observation chamber (Life Imaging Services, Basel, Switzerland). The chamber was placed on an inverted microscope (Nikon Ti-E Eclipse) equipped with an EMCCD camera (Evolve, Roper Scientific, Evry, France), a thermostatic box (Life Imaging Services) providing air at 37 °C and an apochromatic (APO) total internal reflection fluorescence (TIRF) × 100 oil 1.49 numerical aperture (NA) objective. BFP- and GFP-expressing cells were detected using a mercury lamp (Nikon Xcite) and the following filter sets (SemROCK, USA): BFP (excitation: FF01-379/34; dichroic: FF-409Di03; emission: FF01-440/40); EGFP (excitation: FF01-472/30; dichroic: FF-562Di02; emission: FF01-593/40). Cells expressing the different AP constructs, or BFP- or GFP- Nrx1β, were labelled using low concentrations of Atto 594-conjugated mSA, Atto 594-conjugated streptavidin and Atto 594-anti-biotin, or Atto 647N- and 594-conjugated GFP nanobody (1 nM) to isolate single molecules. The GFP nanobody recognizes BFP, given the high sequence homology with GFP. A four-colour laser bench (405; 488; 561; and 642 nm, 100 mW each; Roper Scientific) is connected through an optical fibre to the TIRF illumination arm of the microscope. Laser powers were controlled through acousto-optical tunable filters driven by the Metamorph software (Molecular Devices, USA). Atto 594 and Atto 647N were excited with the 561- and 642-nm laser lines through a four-band beam splitter (BS R405/488/561/635, SemRock). Samples were imaged by oblique laser illumination, allowing the excitation of individual Atto-conjugated ligands bound to the cell surface, without illuminating ligands in solution. Fluorescence was collected using FF01-617/73 and FF01-676/29 nm emission filters (SemRock), respectively, placed on a filter wheel (Suter). Stacks of 2,000–4,000 consecutive frames were obtained from each cell, with an integration time of 20–50 ms. Multicolour fluorescent 100-nm beads (Tetraspeck, Invitrogen) were used to register long-term acquisitions and correct for lateral drifts. In some experiments, EGTA (10 mM) was applied directly in the bath and left for 10 min. For NMDA experiments, a Gilson perfusion system was used to perfuse and change the medium up to 1 h during acquisitions. Neurons were perfused with Tyrode solution containing the fluorescent labels during the whole acquisition time. Cells were recorded in control solution for 10 min, treated with 20 μM NMDA in Tyrode for 10 min and allowed to recover in control solution for 10 min. Control cells were kept in Tyrode solution the whole time. Acquisitions were steered using the Metamorph software (Molecular Devices) in a streaming mode at 50 Hz. Cycles of two consecutive series of acquisitions were made sequentially up to 1 h. One series corresponds to GFP (20 frames, 50 Hz), mSA-Atto 594 (1,500 frames, 50 Hz) and Atto 647N-GFP nanobody (1,500 frames, 50 Hz).

### Trajectory analysis and image reconstruction

Image stacks were analysed using a custom programme running on Metamorph based on wavelet segmentation for localization and simulated annealing algorithms for tracking, described earlier^67^,^68^. The programme allows both the reconstruction of the super-resolution image by summing the positions of localized single molecules into a single image, and tracking of localized molecules through successive images. The instantaneous diffusion coefficient, *D*, was calculated for each trajectory from linear fits of the first 4 points of the mean square displacement (MSD) function versus time. Slow trajectories were defined as trajectories with diffusion coefficients below 0.0093∼0.01 μm^2 ^s^−1^ (Supplementary Fig. 4). This threshold corresponds to molecules exploring a region smaller than that defined by the spatial resolution of the system (∼0.054 μm, full-width at half-maximum) during the time used to fit the initial slope of the MSD (4 points, 20 ms) and is given by *D*_threshold_=(0.054 μm)^2^/(4 × 4 × 0.02 s)∼0.0093 μm^2 ^s^−1^, as described earlier^16^. Spatial resolution was determined using fixed Atto samples. Overall, 415 two-dimensional (2D) distributions of single-molecule positions belonging to long trajectories (>10 frames) were measured by bi-dimensional Gaussian fitting and the resolution determined as 2.3*σ*_*xy*_, where *σ*_*xy*_ is the pointing accuracy. For trajectory analysis, synapses were identified by wavelet-based image segmentation of the Homer1c-GFP postsynaptic marker. The corresponding binary masks were used to sort single-particle data analyses to specific synaptic regions. For NMDA experiments, each mSA and nanobody sequence was analysed separately as described, and data were pooled over 10-min intervals, to obtain representative trajectories of protein populations. The percentage of synapses containing Nlg1 was defined as the ratio between synapses containing at least 10 detections of AP-Nlg1 over the total number of synapses on super-resolved images obtained in the same conditions for mSA and biotin antibody. Synaptic coverage was determined from super-resolved detection maps as the ratio between threshold areas containing detections over a whole synaptic region determined from the low-resolution Homer1c signal. The percentage of synaptic detection is defined as the number of detections within synapses defined by Homer1c divided by the total number of detections. For dual-colour imaging of Nlg1 and Nrx1β, areas with high signal density were identified as domains by wavelet segmentation. Their number and size were extracted from 2D isotropic Gaussian fitting, and their length was determined as the full-width at half-maximum. In the supplemental figures, the number of objects per frame was analysed using the ‘Analyse Particles' plugin in ImageJ and averaged per stack of 2,000 frames acquired every 50 ms, and the number of trajectories per 10 μm was determined as the average number of trajectories per dendritic segments of 10 μm. MSD analysis was performed by filtering trajectories with *D*<0.01 or *D*>0.01 μm^2 ^s^−1^ (Supplementary Fig. 4). The MSD curve as a function of time for *D*<0.1 μm^2 ^s^−1^ was fitted by one-phase association curve on GraphPad to extract the confinement area, which was estimated to be ∼118 nm.

### dSTORM

Primary cultured neurons co-expressing Homer1c-GFP, BirA^ER^ and AP-Nlg1 or CA1 neurons in organotypic slice cultures from Nlg1 KO mice that were single-cell-electroporated with GFP, BirA^ER^ and AP-Nlg1 were surface-labelled with a high concentration (100 nM) of mSA-Alexa647, biotin antibody-Alexa647 or streptavidin-Alexa647 in Tyrode solution for 10 min, and were rinsed and fixed with 4% PFA–0.2% glutaraldehyde in PBS–BSA 1% for 10 min at room temperature or 2 h at 4 °C. dSTORM imaging of cultured neurons and astigmatic-based 3D dSTORM imaging of organotypic brain slices on neurons located close to the coverslip surface was performed. We used an inverted motorized microscope (Nikon Ti, Japan) equipped with a × 100 1.49NA PL-APO objective and a perfect focus system, allowing long acquisition in oblique illumination mode. Both the ensemble and single-molecule fluorescence were collected by using a quad-band dichroic filter (Di01-R405/488/561/635, Semrock). The fluorescence was collected using a sensitive EMCCD (Evolve, Photometrics, USA). For 3D imaging in organotypic brain slices, astigmatism was applied using an adaptive optics system (MicAO—Imagine Optic). 3D calibration was established using 100-nm fluorescent beads (Tetraspeck, Life Technologies) adhered to the slice surface. Single-molecule localization and reconstruction was performed online with automatic feedback control of the lasers using WaveTracer module, enabling optimal single-molecule density during the acquisition^68^. The acquisition and localization sequences were driven by MetaMorph (Molecular Devices) in a streaming mode at 50 frames per second (20-ms exposure time) using an area equal to or less than 256 × 256 pixel region of interest. The brain slice was mounted in an oxygen-scavenging imaging buffer^6^ and sealed between two glass coverslips. Images were composed of 1,038,506 localizations analysed from 64,000 frames, over which sample drift was corrected by localizing and tracking the displacement of cellular autofluorescence. Super-resolution reconstructions were generated with the VISP software^69^, and detection density maps were generated with a neighbourhood radius of 400 nm. The resulting lateral resolution is 20 nm for primary neuronal cultures and 50 nm for slice cultures, while the axial resolution is ∼50 nm. The number of clusters per μm^2^ was determined by wavelet segmentation based on areas with strong signal intensity compared with neighbouring areas^67^,^68^ on the super-resolved dSTORM images generated from 40,000 frames. Synaptic enrichment was defined as the ratio between the average number of synaptic detections (area given by the Homer1c-GFP signal) and the average number of extrasynaptic detections. The dispersion of Nlg1 and LRRTM2 molecules compared with Homer centroid was computed as the distribution of the distances between each detection event and the Homer centroid. Enrichment domains were defined by wavelet segmentation of areas with higher labelling densities relatively to neighbouring environment as illustrated by the linescans in Fig. 7, and their number per synapse was counted for LRRTM2 and Nlg1. Domain sizes were extracted from 2D isotropic Gaussian fittings as the average full-width at half-maximum.

### STED imaging and data analysis

CA1 neurons co-expressing EGFP, BirA^ER^ and AP-Nlg1 in organotypic slices from wild-type mice were surface-labelled with high concentration of mSA-Atto647N (100 mM) in Tyrode solution for 10 min, rinsed, observed live using a commercial STED microscope (TCS SP5, Leica) and thermostated to 37 °C. STED illumination of Atto 647N was performed using a 633-nm pulsed laser providing excitation, and a pulsed bi-photon laser (Mai Tai; Spectra-Physics) tuned to 765 nm and going through a 100-m optical fibre to enlarge pulse width (100 ps) used for depletion. A doughnut-shaped laser beam was achieved through two lambda plates. Fluorescence light between 650 and 740 nm was collected using a photomultiplier, using a HCX PL-APO CS × 100/1.40 NA oil objective and a pinhole open to one time the Airy disk (60 μm). A fivefold zoomed area of 512 × 512 pixels, corresponding to a pixel size of 30 nm, was scanned at 50 Hz. In-depth acquisitions were performed by scanning hippocampal slices with an increment of 1 μm in *z* over 60 μm. When indicated, raw data were deconvoluted using the measured point spread function of the system and the Richardson–Lucy algorithm with Huygens Professional (Huygens Software). 3D reconstructed movies were made using Imaris (Bitplane).

### FRAP experiments and analysis

Neurons electroporated with Homer1c-GFP, BirA^ER^ and AP-Nlg1 or AP-LRRTM2 and cultured for 2 weeks were labelled with high concentration of mSA-Atto594 (100 nM) for 10 min, and then rinsed, mounted in Tyrode solution and observed under the same set-up used for uPAINT. The laser bench (comprising 488, 561 and 642 nm lasers, 100 mW each, Roper Scientific) has a second optical fibre output connected to an illumination device containing galvanometric scanning mirrors (ILAS, Roper Instrument) steered by MetaMorph. It allows precise spatial and temporal control of the focused laser beam at any user-selected region of interest within the sample for targeted photobleaching. Switching between the two fibres for alternating between imaging and bleaching is performed in the millisecond range using a mirror. Oblique illumination acquisition was performed using the 561-nm laser at low power (300 μW at the front of the objective) to precisely image molecules accumulated at the substrate level. After acquiring a 10-s baseline at 0.5–1 Hz frame rate, rapid selective photobleaching of two to three synapses was achieved at higher laser power (3 mW at the front of the objective) during 200–300 ms. Fluorescence recovery was then recorded immediately after the bleach sequence for 30 min at a 0.5–1 Hz frame rate. Observational photobleaching was kept very low, as assessed by observing control synapses nearby. Data were plotted as normalized fluorescence intensity versus time and fitted by the formula^44^: *φ* [1−erf(1/(2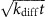)]+(1−φ)[1−exp(−*k*_reac_*t*)], where *φ* is the fraction of synaptic Nlg1 or LRRMT2 molecules diffusing freely with coefficient *D*, *k*_diff_ (in min^−1^) is a characteristic diffusive rate equal to *D*/*r*^2^ (where *r* is the radius of the bleached area), (1−*φ*) the fraction of molecules trapped in adhesive interactions with presynaptic Nrx1β and *k*_reac_ (in min^−1^) the turnover rate of bound molecules. In this model, the synaptic enrichment of Nlg1 or LRRTM2 at synapses is equal to the total number of molecules (bound + free) versus free molecules, that is, the ratio 1/*φ*. In control experiments, FRAP was performed on DIV 15 neurons electroporated with Nlg1-GFP using a 488-nm laser power of either 0.4 or 4 mW at the front of the objective.

### Confocal microscopy

Single electroporated neurons from organotypic slice culture co-expressing GFP, BirA^ER^ and AP-Nlg1 were surface-labelled with mSA-Atto 647N, biotin antibody-Atto594 or streptavidin-Atto 647N in artificial cerebrospinal fluid (ACSF) (in mM, 125 NaCl, 2.5 KCl, 2 CaCl_2_, 1 MgCl_2_, 25 NaHCO_3_, 1.25 NaH_2_PO_4_ and 25 glucose, pH 7.4) for 10 min or 1 h, rinsed and fixed with 4% PFA–0.2% glutaraldehyde in PBS–BSA 1% for 2 h at 4 °C. Images were acquired on a commercial Leica DMI6000 TCS SP5 microscope using a × 63, 1.4 NA oil objective and a pinhole opened to one time the Airy disk. Images of 512 × 512 pixels were acquired at a scanning frequency of 400 Hz.

### Statistics

Statistical values are given as mean±s.e.m., unless otherwise stated. Statistical significance was calculated using GraphPad Prism. All data sets comparing two conditions were tested by the non-parametric Mann–Whitney test. Data sets containing more than two conditions were compared by one-way analysis of variance test, followed by a *post hoc* Dunn's test. Sample size was based on two to three different cultures per condition, 2–10 cells per experiment. Randomization of samples was performed for all experiments. When critical comparison of different labelling conditions was involved, experiments and image analysis were performed blindly.

## 

## Additional information

**How to cite this article:** Chamma, I. *et al*. Mapping the dynamics and nanoscale organization of synaptic adhesion proteins using monomeric streptavidin. *Nat. Commun.* 7:10773 doi: 10.1038/ncomms10773 (2016).

## Supplementary Material

Supplementary Figures Supplementary Figures 1-11

Supplementary Movie 1 mSA is washed off the cell surface by addition of free biotin. COS cells co-expressing GFP, AP-SEP-TfR, and BirAER were labeled with mSA-Atto 647N, and single mSA molecules bound to membrane-expressed AP-Nlg1 were tracked with uPAINT. A time lapse movie of 10 min was recorded at a rate of one image every 2 sec. 200 μM biotin was added after 2 min (white square). The movie shows the merge between the GFP image taken at the beginning of the recording (red), and single mSA-Atto 647N molecules tracked over time (green dots). Note the rapid decline in the number of mSA molecules after addition of biotin, reflecting their detachment from biotinylated AP-Nlg1 molecules by competition with excess free biotin.

Supplementary Movie 2 Neuroligin-1 diffusion on the dendritic membrane and accumulation at synapses. DIV15 neurons co-expressing the synaptic marker Homer1c-GFP, AP-Nlg1, and BirAER were labeled with mSA-Atto 594, and single mSA molecules binding to AP-Nlg1 expressed at the cell surface were tracked by uPAINT. The movie represents 20 s (400 planes of 50 ms each). AP-Nlg1 is shown as red dots diffusing on the dendritic surface (dotted contour), and at synapses (white). Note the slow diffusion of Nlg1 at synapses.

Supplementary Movie 3 STED imaging of neuroligin-1 in organotypic brain slices. Overlay of confocal (GFP) and STED (red) stacks in z (1 plane every 1 μm) of single cell electroporated neurons expressing GFP, AP-Nlg1, and BirAER, in a mouse organotypic brain slice. Note the high specificity of mSA labeling, and extremely low background.

Supplementary Movie 4 STED imaging of neuroligin-1 accumulation in dendritic spines. High magnification deconvolved STED (red) and confocal (GFP) 3D animations from a dendritic portion of a single cell electroporated neuron expressing GFP, AP-Nlg1, and BirAER, in a mouse organotypic brain slice. Note the strong labeling of Nlg1 in dendritic spines.

Supplementary Movie 5 3D dSTORM imaging of AP-Nlg1 in a Nlg1 knock-out organotypic brain slice. Astigmatic-based 3D dSTORM reconstructed movie of a dendritic segment from a CA1 neuron expressing AP-Nlg1, BirAER, and EGFP, within an organotypic brain slice from Nlg1 KO mice. The image is color-coded with respect to the detection density, and shows the enrichment of Nlg1 in dendritic spines.

Supplementary Movie 6 Dual-color imaging of neurexin-1β and neuroligin-1 at synapses. Neurons electroporated with either Homer1c-GFP, AP-Nlg1, and BirAER, or BFP-Nrx1β, were plated together and cultured until DIV15. AP-Nlg1 was labeled with mSA-Atto 594, and BFP-Nrx1β with Atto-647N-nanobody, simultaneously on the imaging setup. Sequential acquisitions of mSA-Atto 594 and Atto 647N-nanobody were performed by uPAINT, and superimposed here for a time period of 20 s (400 planes of 50 ms each). The movie represents the overlay of mSA-Atto594 (red), Atto 647N-nanobody (green), and Homer1c-GFP (white). The dotted line shows the contour of a dendrite expressing Homer1c-GFP, AP-Nlg1, and BirAER.
