# Supplementary material for: Mapping the dynamics and nanoscale organization of synaptic adhesion proteins using monomeric streptavidin
Source: Nat Commun. 2016 Mar 16;7:10773. doi: 10.1038/ncomms10773 (PMC4799371; doi:10.1038/ncomms10773)
Supplement: Supplementary Figures — 1-11 [file ncomms10773-s1.pdf]

a

mSA sequence (MW 15.5 kDa)

```
  1 MASHHHHHHHHHHENLYFQSGSAEAGITGTWYNQSGSTFTVTAGADGNLT
 51 GQYENRAQGTGCQNSPYTLTGRYNGTKLEWRVEWNNSTENCHSRTEWRGQ
101 YQGGAEARINTQWNLTYEGGSGPATEQGQDTFTKVKPSAA
```

anti-GFP nanobody sequence (MW 15.3 kDa)

```
  1 MADVQLVESGGALVQPGGSLRLSCAASGFPVNRYSMRWYRQAPGKEREWV
 51 AGMSSAGDRSSYEDSVKGRFTISRDDARNTVYLQMNSLKPEDTAVYYCNV
101 NVGFEYWGQGTQVTVSSADPNSSSVDKLAAALEHHHHHH
```

b

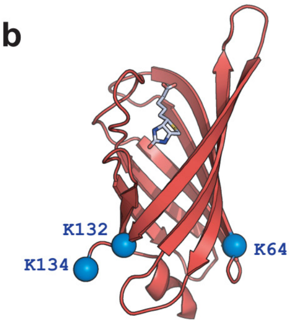

c

Labeled protein	MW	Estimated DOL
mSA-Atto 647N	15 kDa	0.5
mSA-Atto 594	15 kDa	1.2
mSA-Alexa 647	15 kDa	2.1
Atto 647N-nanobody	15 kDa	0.6
Strept-Atto 594	75 kDa	2.3
Strept-Atto 647N	75 kDa	2.2
Anti-biotin-Atto 594	150 kDa	8
Anti-biotin-Alexa 647	150 kDa	1.1

d

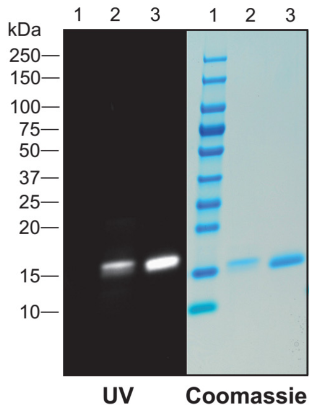

e

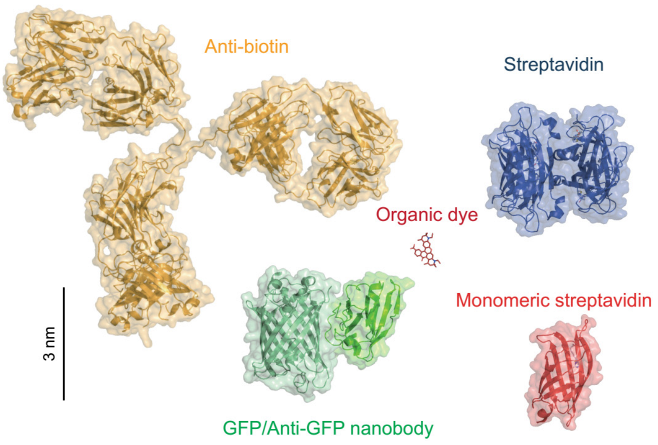

Supplementary Figure 1. Label production and fluorophore conjugation

(a) Sequences of mSA and GFP nanobody with lysine residues in bold blue. **(b)** Structure of mSA (adapted from PDB entry 4JNJ), with lysines highlighted in blue and the biotin in stick representation. **(c)** Degree of labeling (DOL) obtained for all the conjugated proteins used in the study, except for streptavidin-Alexa647 which was purchased. **(d)** Illustrative SDS-PAGE of conjugated mSA and GFP nanobody. Left, fluorescence image obtained with UV illumination and emission filtered at 655 nm; right, Coomassie staining of the corresponding gel. 1: molecular weight ladder; 2: mSA-Atto 594; 3: Atto 647N-GFP nanobody. **(e)** Size comparison between biotin antibody (adapted from PDB entry 1IGT), tetrameric streptavidin (adapted from PDB entry 3RY2), monomeric streptavidin (adapted from PDB entry 4JNJ), GFP and anti-GFP Nanobody complex (PDB entry 3G9A), and an Atto dye.

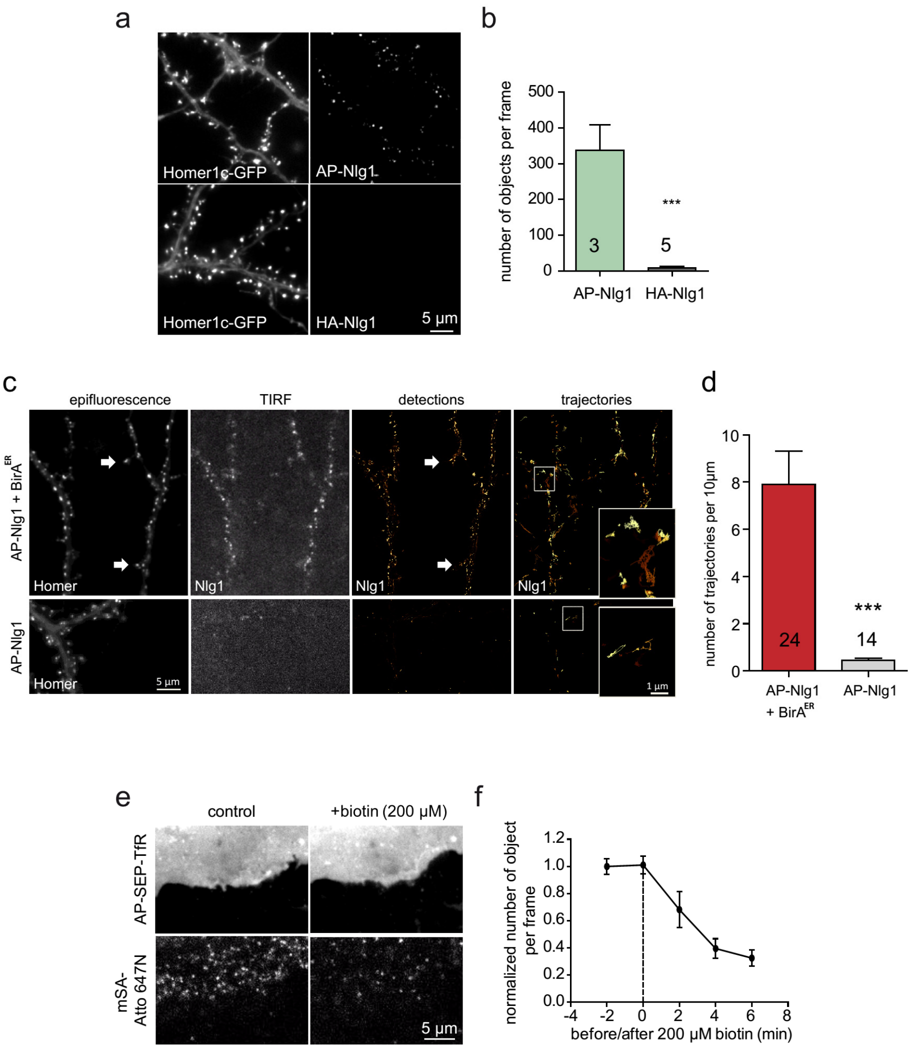

Supplementary Figure 2. mSA specifically labels biotinylated AP-tagged proteins

(a) DIV15 hippocampal neurons were electroporated with the synaptic marker Homer1c-GFP, AP-Nlg1 or non biotinylatable HA-Nlg1, plus BirA^{ER}. Biotinylated AP-Nlg1 was successfully labeled with mSA-Atto 594 at the cell surface (top right), whereas no labeling of HA-Nlg1 was detected (bottom right). **(b)** Mean number of single molecules detected per frame from neurons expressing AP-Nlg1 or HA-Nlg1 (***, $p < 0.0001$. data are from one experiment). **(c)** DIV 15 hippocampal neurons electroporated with Homer1c-GFP, AP-Nlg1, and with (top) or without (bottom) BirA^{ER}. AP-Nlg1 was successfully labeled with mSA-Atto 594 in the presence of BirA^{ER} but no labeling was detected when BirA^{ER} was omitted. **(d)** Mean number of single molecules per frame from neurons expressing AP-Nlg1 and BirA^{ER} or AP-Nlg1 alone (***, $p < 0.0001$. Data are from 2 different cultures). **(e)** COS-7 cells expressing AP-tagged SEP-transferrin receptor (AP-SEP-TfR) were labeled with mSA-Atto 647N. Bound mSA was washed off with 200 µM biotin, showing the specificity and reversibility of labeling. **(f)** Normalized labeling density of mSA-Atto 647N stained cells before and after addition of excess biotin (n = 4 from 2 different experiments, mean ± sem).

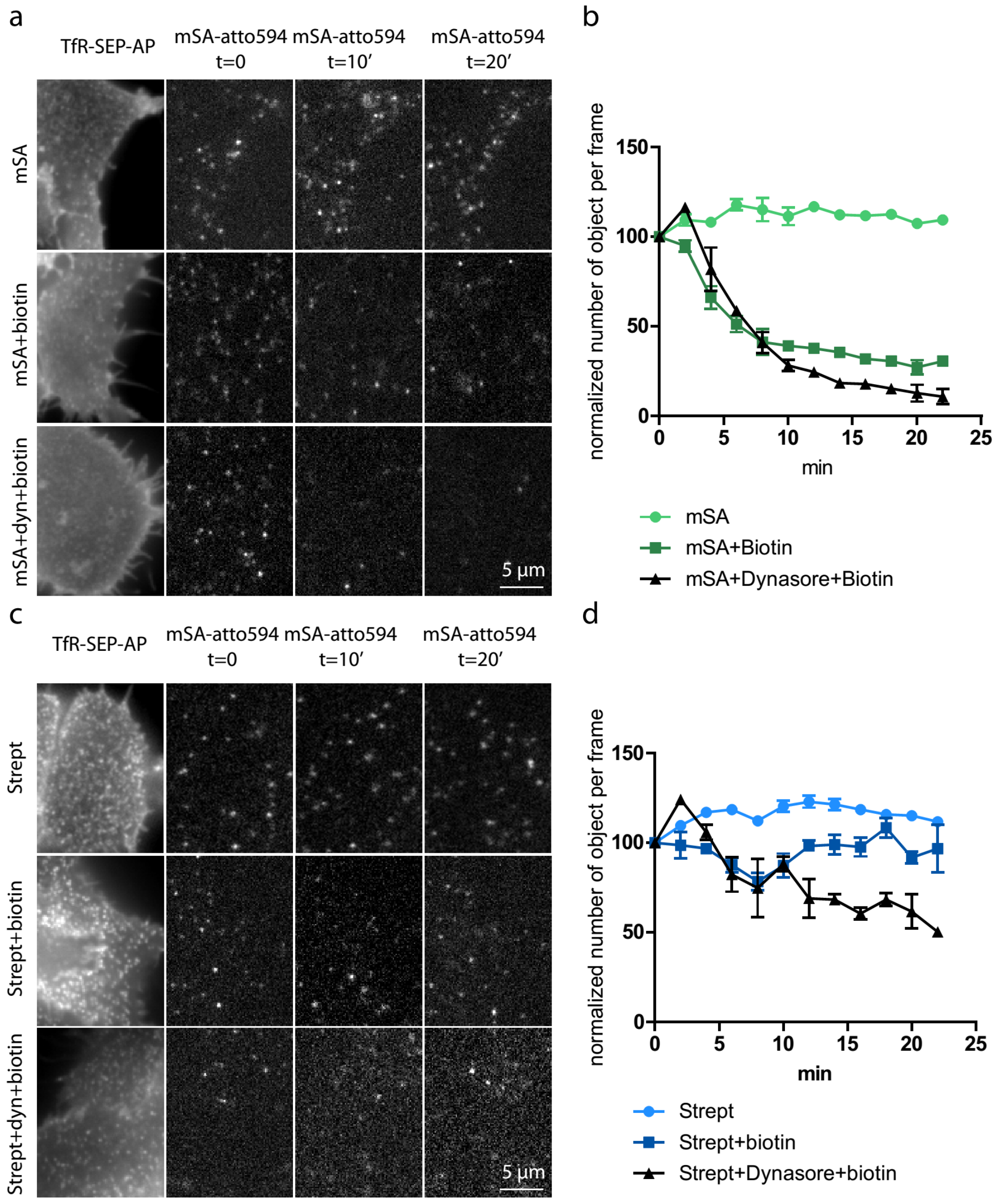

Suppplementary Figure 3. Biotin washout of surface mSA to access intracellular compartments

(a, c) HEK-293 cells expressing the transferrin receptor AP-TfR-SEP and BirA^{ER} were labeled with mSA-Atto594 (a) or streptavidin-Atto594 (c). Stacks of 100 frames at a 50 ms exposure time were acquired every 2 min for 22 min, and the number of detected particles per stack was averaged per frame and normalized to the first acquisition (t = 0). Where indicated, 50 µM dynasore was added to the imaging medium at t = -10 min to inhibit endocytosis, and 200 µM non permeable biotin was added at t = 3 min. **(b, d)** Normalized mean particle per frame detected with mSA (b) or streptavidin (d) in control (mSA, n=3; Strept, n=2), biotin (mSA, n=10; Strept, n=4), or dynasore and biotin (mSA, n=8; Strept, n=2), over time. Biotin efficiently washed out the extracellular mSA signal coupled to TfR-SEP-AP (a, b), but not the streptavidin signal (c, d), allowing us to visualize internalized proteins. Data are from 2 different experiments.

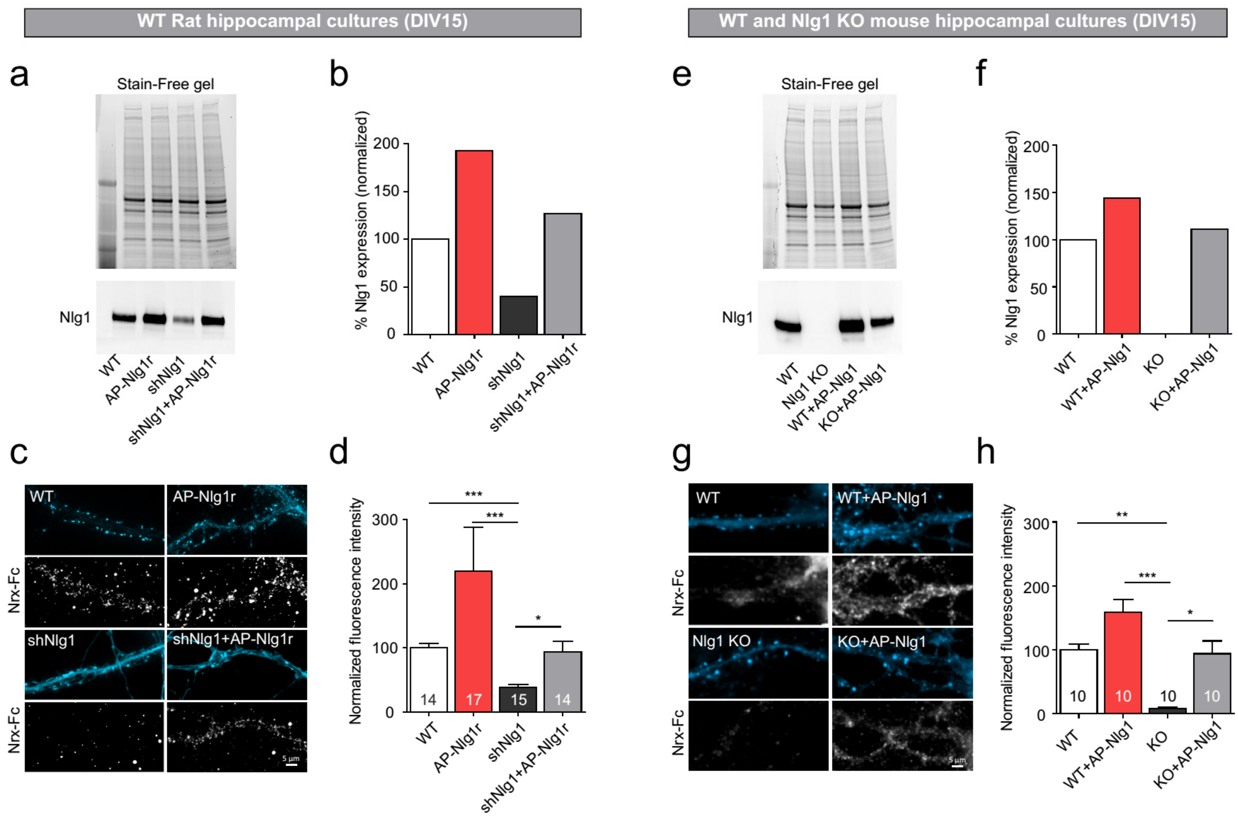

Supplementary Figure 4. Overexpression and rescue of AP-Nlg1 in rat and mouse hippocampal neurons **(a-d)** Rat hippocampal neurons were electroporated at DIV0 either with an empty vector, AP-Nlg1r, shNlg1, or shNlg1 + AP-Nlg1r and processed at DIV15. **(a)** Anti-Nlg1 immunoblot revealing the Nlg1 protein level in the 4 different conditions, the above stain-free gel serving as a control of sample loading. **(b)** Quantification of Nlg1 levels, normalized to the condition of neurons expressing empty vector. **(c)** In parallel coverslips, neurons were live surface labeled with soluble Nrx1β-Fc, fixed, and immuno-stained with Cy5-conjugated anti-human-Fc antibody. **(d)** Normalized Cy5 fluorescence intensity in the 4 conditions**. (e-h)** Mouse hippocampal neurons from wild-type or Nlg1 KO mice were electroporated at DIV0 either with an empty vector or AP-Nlg1 and processed at DIV15**. (e)** Western blot showing the levels of Nlg1 protein in the 4 conditions and corresponding stain-free gel. **(f)** Normalized Nlg1 protein levels. **(g)** Representative images of Nrx1β binding in the 4 conditions. **(h)** Nlg1 membrane expression quantified using the Nrx1β binding assay in the same set of experiment. A ratio of two in the Nrx1β-Fc signal between cells expressing AP-Nlg1 and cells not expressing AP-Nlg1 indicates that there is roughly one copy of AP-Nlg1 for one copy of endogenous Nlg1. Data are from one experiment for each condition.

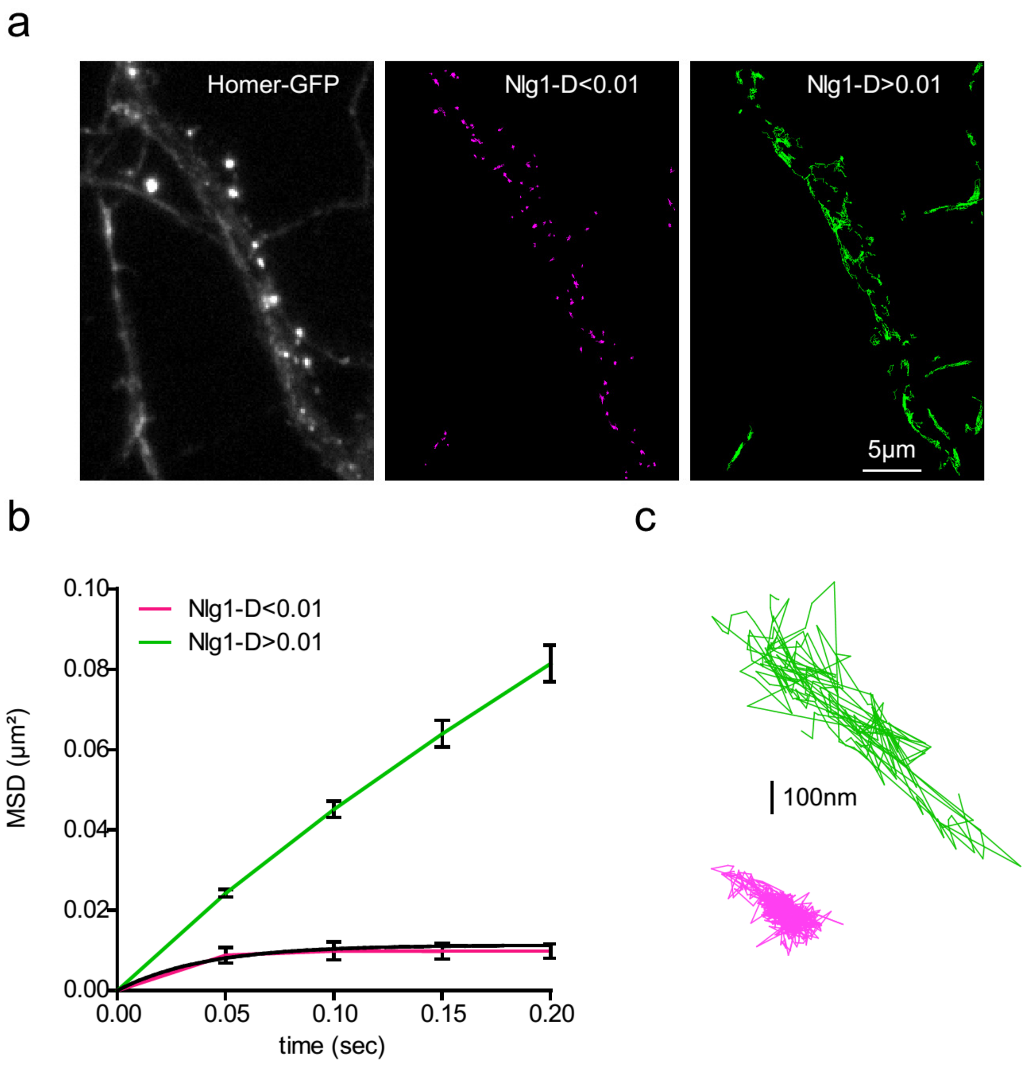

Supplementary Figure 5. Quantitative discrimination between slow and fast diffusing molecules
(a) DIV 15 hippocampal neurons expressing Homer1c-GFP, AP-Nlg1, and $BirA^{ER}$ were labeled with mSA-Atto594 and AP-Nlg1 molecules tracked by uPAINT. Diffusion coefficients inferior to 0.01 $\mu m^2 s^{-1}$ (defined as the threshold for slow trajectories, see material and methods) are represented in magenta, and diffusion coefficients superior to 0.01μm^2/s are shown in green. **(b)** Average mean squared displacement (MSD) over time for trajectories with D > 0.01 $\mu m^2 s^{-1}$ (linear, green, n=4, 2411 trajectories), and D < 0.01 $\mu m^2 s^{-1}$ (confined, magenta, n=4, 2112 trajectories). The diffusive MSD curve was fitted by a linear equation MSD(t) = 4Dt. The confined MSD curve was fitted to the one-phase exponential equation MSD(t) = (L^2/3)(1-exp(-t/τ)), where L is the confinement diameter and τ is a time constant [69]. The confinement diameter (L) was determined to be ~118 nm. **(c)** Representative trajectories with D > 0.01$\mu m^2 s^{-1}$ (green) and D < 0.01$\mu m^2 s^{-1}$ (magenta). Data are from 3 different experiments.

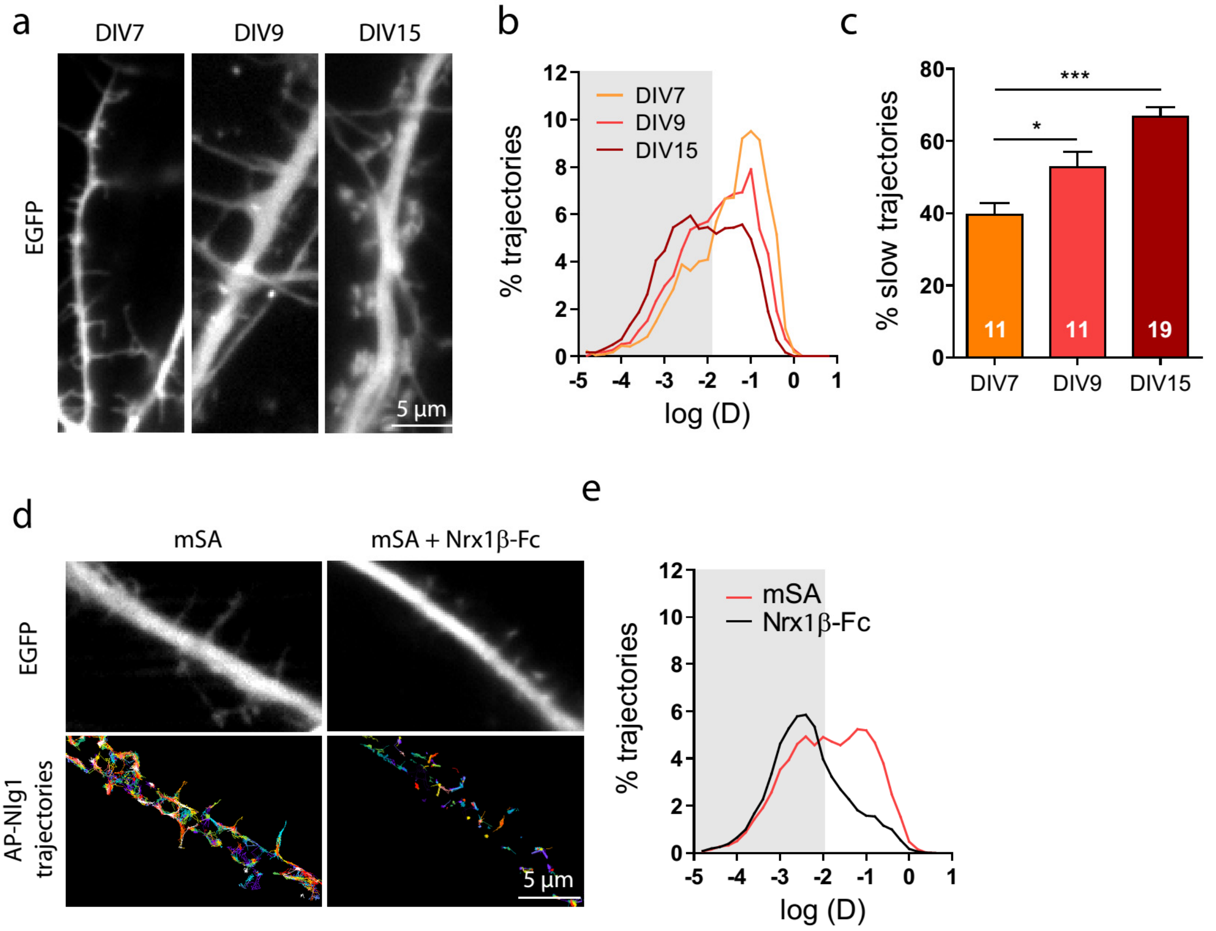

Supplementary Figure 6. Age-dependent decrease of Nlg1 diffusion and stabilizing effect of Nrx1β binding

(a) EGFP signal in DIV 7, 9 or 15 neurons expressing EGFP, AP-Nlg1, and $BirA^{ER}$ and showing the progressive maturation of dendritic filopodia into spines. **(b)** Distribution of AP-Nlg1 diffusion coefficients across neuronal development (DIV7, n=11; DIV9, n=11; DIV15, n=19 from 2 different experiments). Note the progressive reduction in diffusion coefficient as neurons grow older. **(c)** Corresponding percentage of slow trajectories with $D < 0.01\ \mu m^2\ s^{-1}$ (*, $p < 0.05$; ***, $p < 0.0001$). **(d)** EGFP images of dendritic segments of DIV 15 neurons and corresponding trajectories of AP-Nlg1 labeled with mSA-Atto594, upon acute addition of vehicle or Nrx1β-Fc dimers. **(e)** Corresponding distribution of AP-Nlg1 diffusion coefficients in the two conditions (mSA, n=12; mSA + Nrx1β-Fc, n=7 cells from 2 different experiments). Note that addition of Nrx1β-Fc reduces AP-Nlg1 diffusion, indicating that insertion of the AP tag does not alter Nlg1 binding to Nrx1β, and confirming the previously identified mechanism that Nrx1β binding to Nlg1 triggers the synaptic anchorage of Nlg1 via PSD-95 [31].

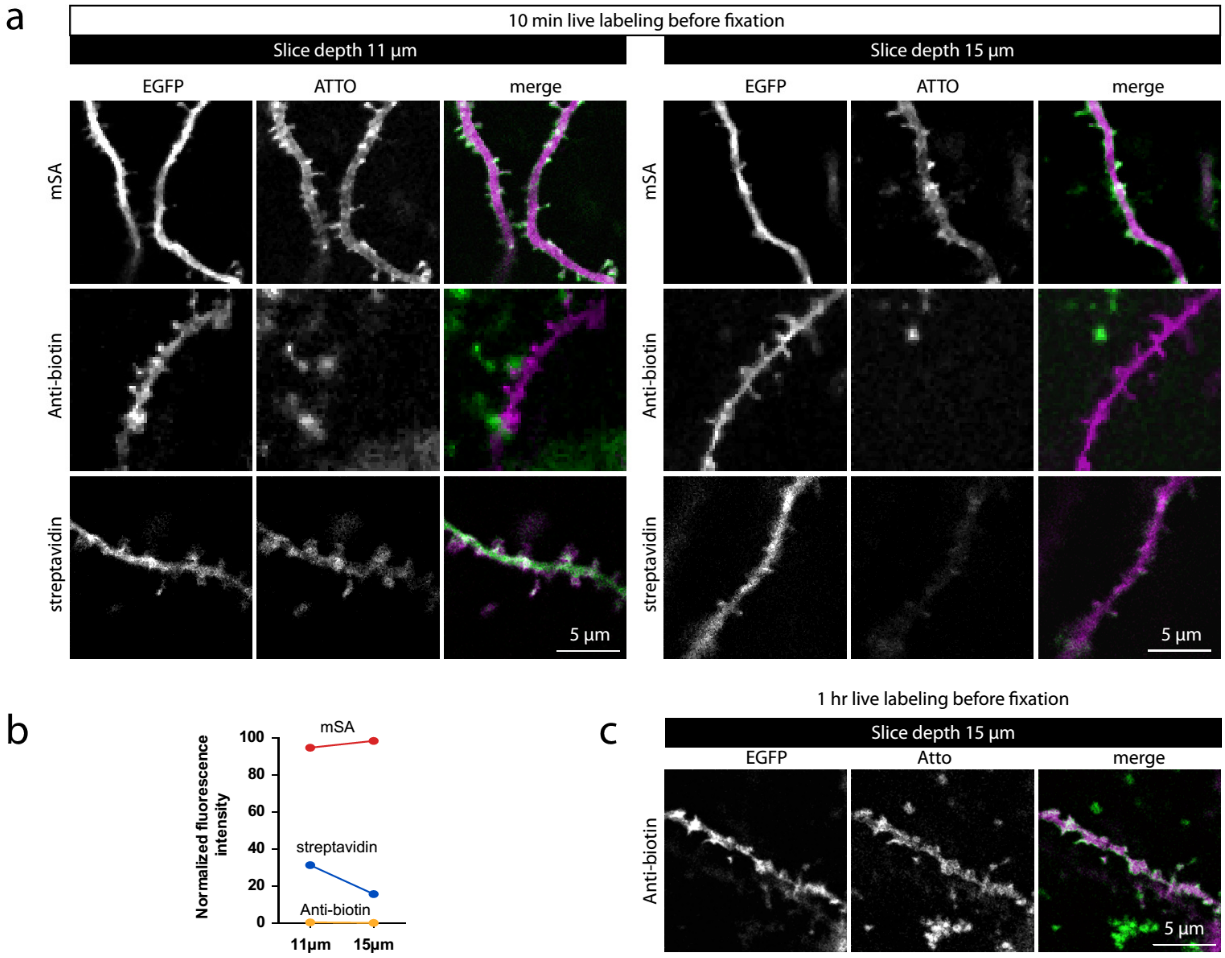

Supplementary Figure 7. mSA efficiently labels Nlg1 within deep and confined tissue environment

(a) Live neurons from organotypic hippocampal slices electroporated with EGFP, AP-Nlg1, and BirA^{ER}, were labeled for 10 min with mSA-Atto647N (top), anti-biotin-Atto594 (middle), or streptavidin-Atto647N (bottom) before fixation. At 15 µm from slice surface, only mSA efficiently labeled AP-Nlg1, whereas at 11 µm, streptavidin and mSA labeled AP-Nlg1. **(b)** Quantification of the fluorescence intensity in the three different conditions shown in (a) normalized to the GFP intensity and to the degree of labeling of each probe (Supplementary Fig. 1). **(c)** After a 1 hr live labeling, anti-biotin-Atto594 was able to efficiently label AP-Nlg1 at the same sample depth. Data are from 2 slice cultures.

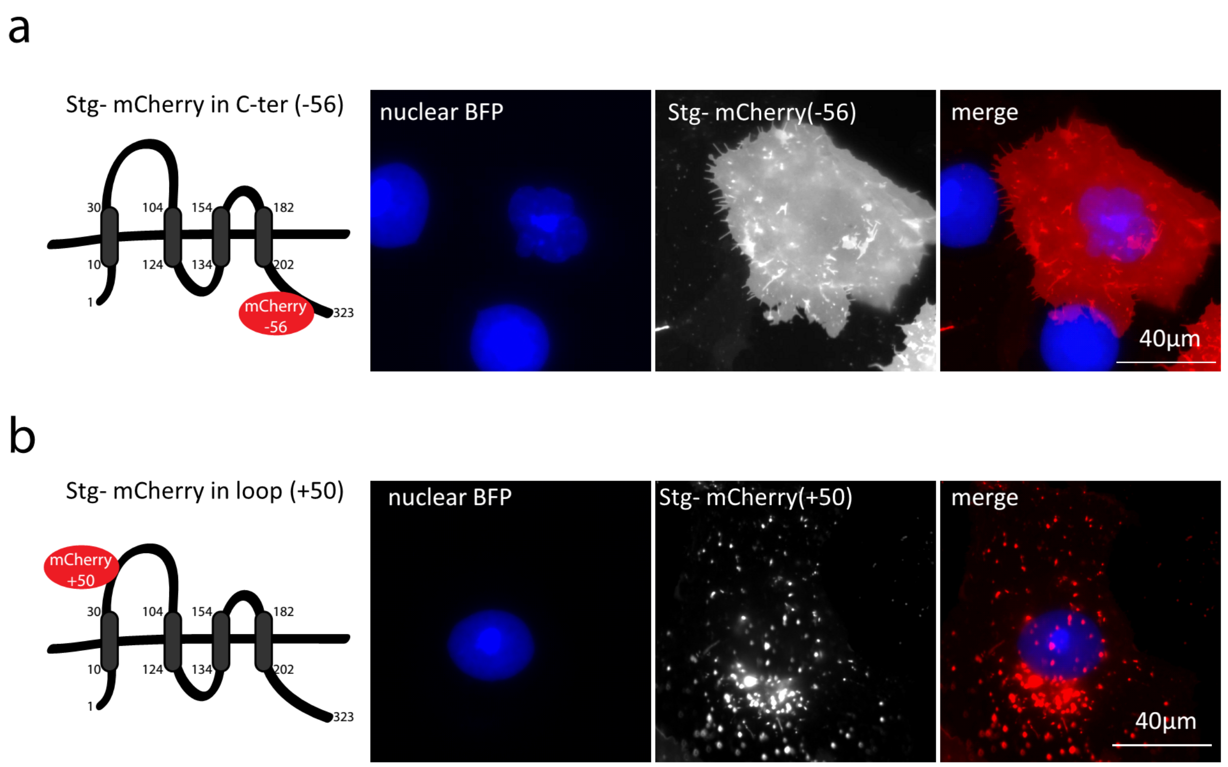

Supplementary Figure 8. Insertion of mCherry in the stargazin extra-cellular loop disrupts the targeting of stargazin to the plasma membrane. (a) When mCherry is inserted at position -56aa before the C-terminus of stargazin, the protein expressed in COS-7 cells is targeted to the cell membrane. **(b)** When mCherry is inserted with the first extracellular loop of stargazin, the protein remains in intracellular compartments. The nucleus is stained with co-expressed nuclear BFP (blue), and the mCherry signal is shown in red.

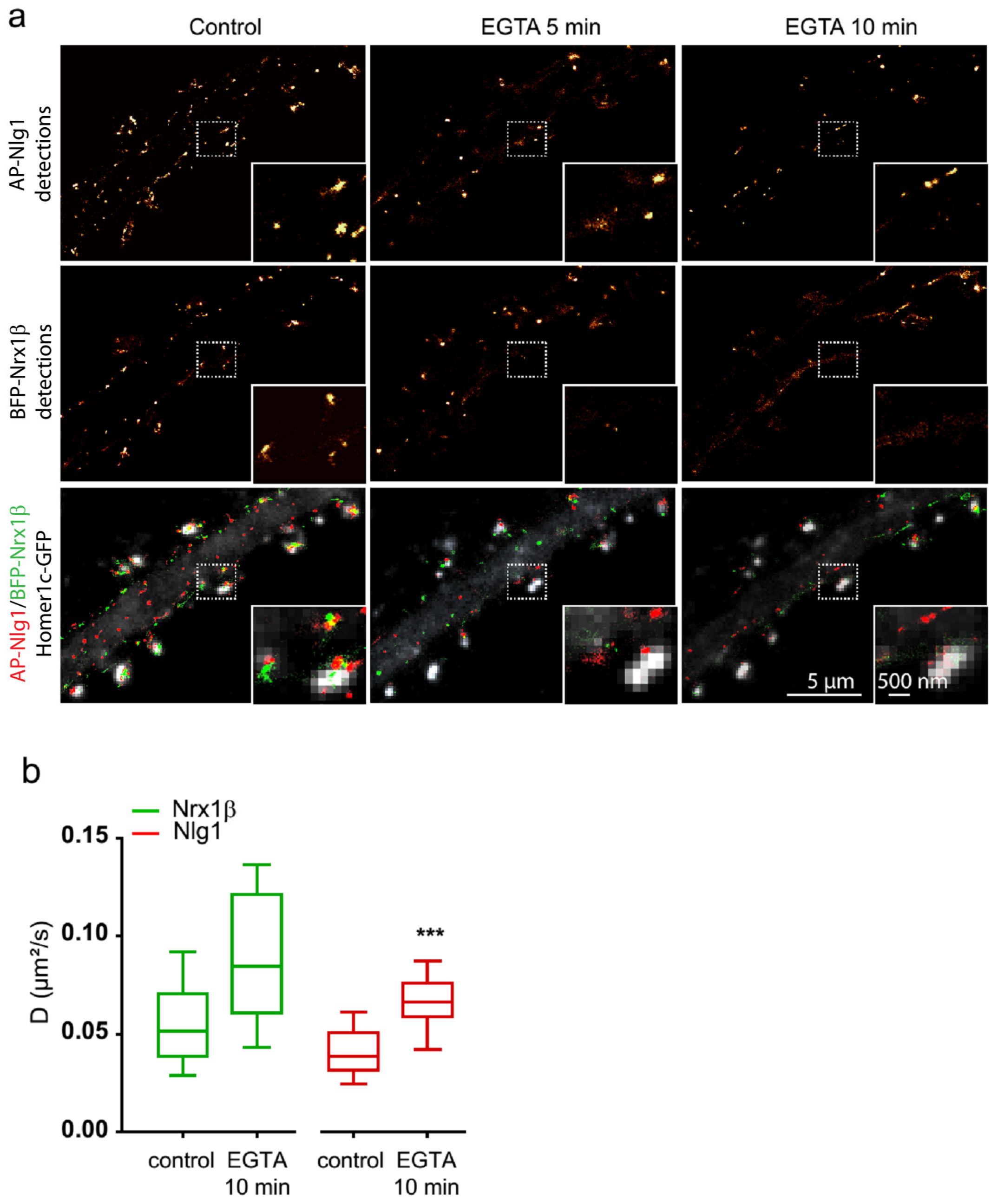

Supplementary Figure 9. Destabilization of trans-synaptic Nrx1β/Nlg1 adhesion by EGTA

(a) Dual-color uPAINT imaging of BFP-Nrx1β and AP-Nlg1 labeled with GFP nanobody-Atto647 and mSA-Atto594, respectively at axon/dendrite contacts of DIV15 neurons. Acute addition of EGTA (10 mM) induced a gradual loss of Nrx1β and Nlg1 from synapses. **(b)** Median ± IQR diffusion coefficient of BFP-Nrx1β (green) and AP-Nlg1 (red) molecules before and after EGTA treatment (n=6 cells for each condition from 2 different experiments, ** $p < 0.01$).

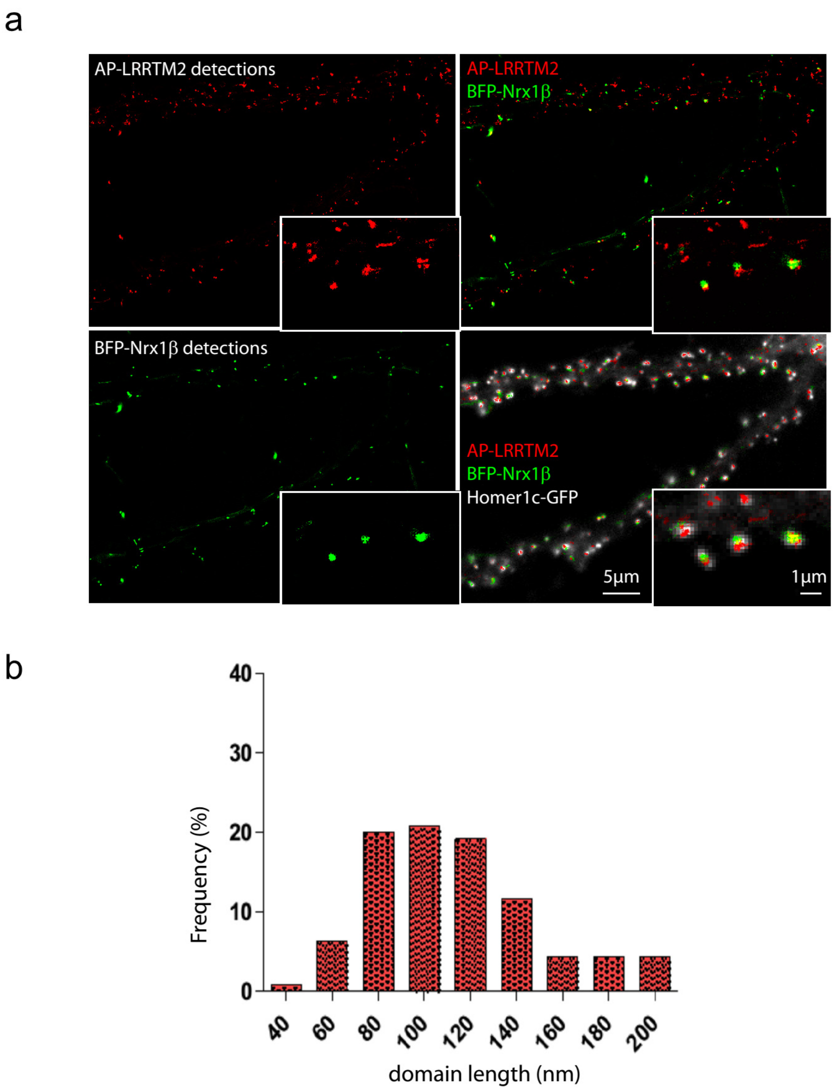

Supplementary Figure 10. Dual-color super resolution imaging of trans-synaptic contacts between Nrx1β and LRRTM2

(a) Neurons co-expressing AP-LRRTM2, BirA^{ER}, and Homer1c-GFP, or expressing BFP-Nrx1β were co-cultured for 15 days and labeled with mSA-Atto 594 and Atto 647N-nanobody. Integrated density of LRRTM2 (red) and Nrx1β (green) molecules at axon/dendrite contacts are shown. **(b)** Size distribution of LRRTM2 nanodomains was measured by uPAINT (n=6 cells from 3 different experiments).

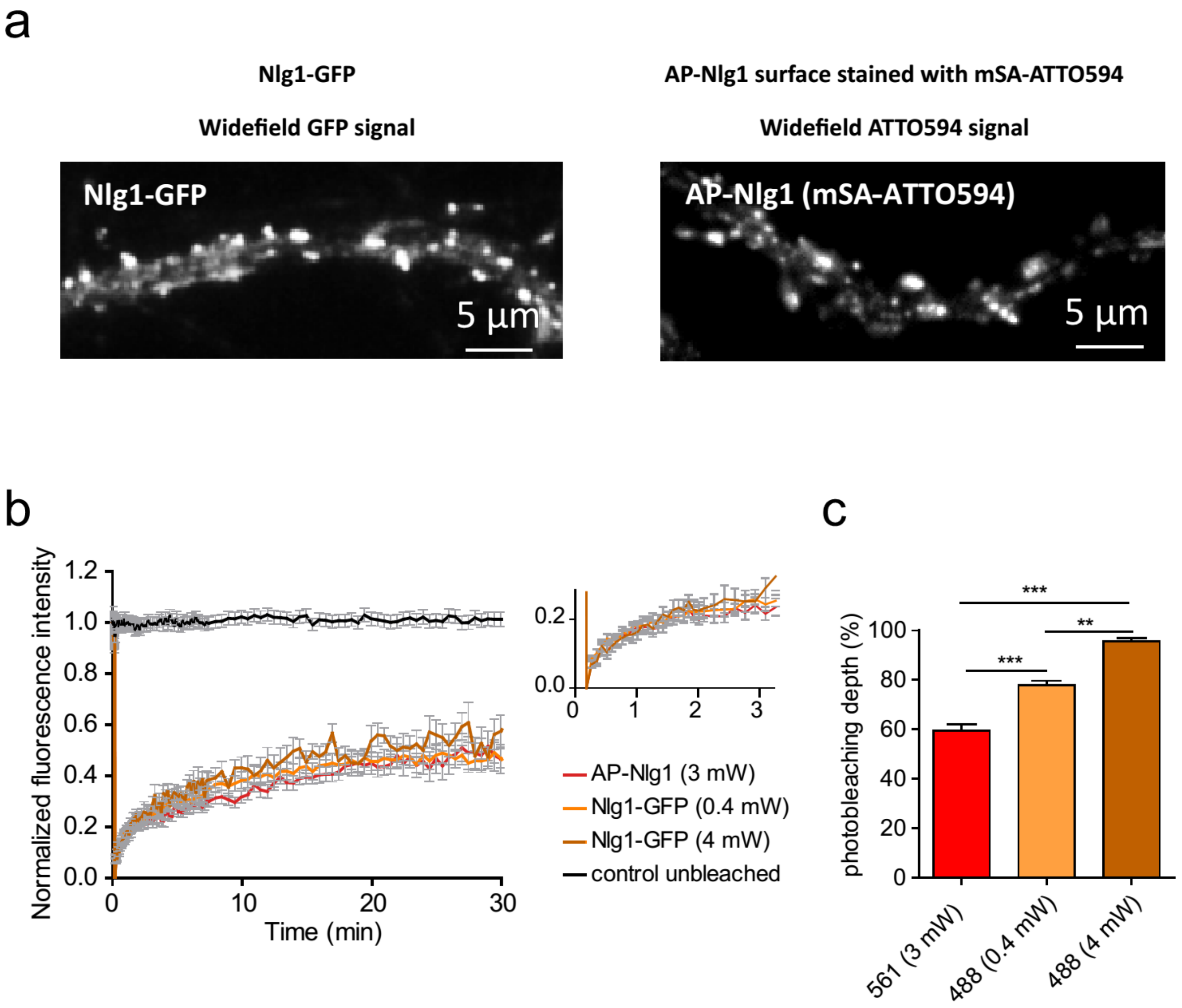

Supplementary Figure 11. Comparison of AP-Nlg1 and Nlg1-GFP dynamics in mature hippocampal neurons by FRAP

(a) Representative wide field images of DIV 15 neurons expressing Nlg1-GFP, or AP-Nlg1, Homer1c-GFP, and BirA^{ER} labeled with mSA-Atto 594. **(b)** FRAP experiments were performed on Nlg1-GFP using a low 488 nm laser power (0.4mW) or a high 488 nm laser power (4mW); and AP-Nlg1 labeled with mSA-Atto594 using a 3 mW 561 nm laser power. Corresponding normalized fluorescence recovery curves are shown. (AP-Nlg1, n=25; Nlg1-GFP (0.4mW), n=27; Nlg1-GFP (4mW), n=11). **(c)** Corresponding percentage of photobleaching in each of the three conditions measured by dividing the mean fluorescent baseline by the intensity after photobleaching (AP-Nlg1, n=25; Nlg1-GFP (0.4mW), n=27; Nlg1-GFP (4mW), n=11; *** $p < 0.0001$, 1-way ANOVA). Data are from 3 different experiments for AP-Nlg1 and 2 different experiments for Nlg1-GFP.
